# Prevalence and correlates of anal intercourse among female sex workers in eSwatini

**DOI:** 10.1371/journal.pone.0228849

**Published:** 2020-02-11

**Authors:** Branwen N. Owen, Mathieu M-Giroux, Sindy Matse, Zandile Mnisi, Stefan Baral, Sosthenes C. Ketende, Rebecca F. Baggaley, Marie-Claude Boily

**Affiliations:** 1 Department of Infectious Disease Epidemiology, Imperial College London, London, United Kingdom; 2 Department of Epidemiology, Biostatistics, and Occupational Health, McGill University, Montréal, Canada; 3 Ministry of Health, Mbabane, eSwatini; 4 Department of Epidemiology, Johns Hopkins School of Public Health, Baltimore, MD, United States of America; 5 MRC Centre for Global Infectious Disease Analysis, Imperial College, London, United Kingdom; University of Westminster, UNITED KINGDOM

## Abstract

**Introduction:**

As HIV is very effectively acquired during condomless receptive anal intercourse (AI) with serodiscordant and viremic partners, the practice could contribute to the high prevalence among female sex workers (FSW) in eSwatini (formerly known as Swaziland). We aim to estimate the proportion reporting AI (AI prevalence) among Swazi FSW and to identify the correlates of AI practice in order to better inform HIV prevention interventions among this population.

**Methods:**

Using respondent-driven sampling (RDS), 325 Swazi FSW were recruited in 2011. We estimated the prevalence of AI and AI with inconsistent condom use in the past month with any partner type, and inconsistent condom use during AI and vaginal intercourse (VI) by partner type. Univariate and multivariable logistic regression models were used to identify behavioural and structural correlates associated with AI and AI with inconsistent condom use.

**Results:**

RDS-adjusted prevalence of AI and AI with inconsistent condom use was high, at 44%[95% confidence interval (95%CI):35–53%]) and 34%[95%CI:26–42%], respectively and did not vary by partner type. HIV prevalence was high in this sample of FSW (70%), but knowledge that AI increases HIV acquisition risk low, with only 10% identifying AI as the riskiest sex act. Those who reported AI were more likely to be better educated (adjusted odds ratio(aOR) = 1.92[95%CI:1.03–3.57]), to have grown up in rural areas (aOR = 1.90[95%CI:1.09–3.32]), have fewer new clients in the past month (aOR = 0.33[95%CI:0.16–0.68]), and for last sex with clients to be condomless (aOR = 2.09[95%CI:1.07–4.08]). Although FSW reporting AI in past month were more likely to have been raped (aOR = 1.95[95%CI:1.05–3.65]) and harassed because of being a sex worker (aOR = 2.09[95%CI:1.16–3.74]), they were also less likely to have ever been blackmailed (aOR = 0.50[95%CI:0.25–0.98]) or been afraid to walk in public places (aOR = 0.46[95%CI:0.25–0.87]). Correlates of AI with inconsistent condom use were similar to those of AI.

**Conclusions:**

AI is commonly practised and condom use is inconsistent among Swazi FSW. Sex act data are needed to determine how frequently AI is practiced. Interventions to address barriers to condom use are needed, as are biomedical interventions that reduce acquisition risk during AI.

## Introduction

eSwatini faces the highest HIV prevalence in the world, with an estimated 34% of 15 to 49 year old women living with HIV[1]. Worldwide, female sex workers (FSW) bear a disproportionately high burden of disease compared to other women of reproductive age in the population[2], and this is no different in eSwatini, where 70% of FSW are estimated to be living with HIV[3]. HIV is very effectively transmitted by condomless anal intercourse with serodiscordant and viremic partners[4], with a meta-analysis on finding that women may have an 18-fold higher HIV acquisition risk during receptive condomless AI compared to condomless vaginal intercourse[5]. Despite anal intercourse (AI) being commonly practiced among FSW in sub-Saharan Africa[6], public health messaging to FSW on HIV transmission frequently neglects safe anal sex practices[7–9]. This neglect may contribute to limited awareness of transmission risk during condomless AI[10], and subsequently to the tendency towards lower rates of condom use during AI compared to vaginal intercourse (VI)[11–13].

AI practice among FSW appears to be associated with other sexual behaviours associated with higher risk of HIV and sexually transmitted infections (STI), including having a greater number of clients[10,14–16], practice of ‘dry’ sex[17,18], condomless sex[18,19] and difficulty negotiating condom use[14]. AI practice is often more common among FSW who suffer physical[17,19–22] or sexual violence[17,18]. Previous research on Swazi FSW points to conditions where AI is likely to be commonly practised and to frequently be condomless[23–26]. Sex work is illegal in eSwatini, and as such is hidden, marginalised and stigmatised[23,24]. Violence, both physical and sexual, is commonly perpetrated against Swazi FSW, but given the legal status of sex work, is rarely reported to the police[24,25], who are themselves frequently the perpetrators[26]. Most FSW report wanting to use condoms consistently, but structural factors, including financial incentives, act as barriers to condom use[23,24].

Using a national cross-sectional survey among FSW eSwatini, we aim 1)to estimate the proportion reporting AI and AI with inconsistent condom use (which we refer to as AI prevalence and AI prevalence with inconsistent condom use, respectively), 2)to compare condom use during AI and VI by partner type, and 3)to identify the correlates of AI practice. Such information is necessary to tailor appropriate HIV prevention interventions for FSW in eSwatini and other southern African countries.

## Methods

### Study design and population

From July to September 2011, 325 Swazi FSW were recruited using respondent-driven sampling (RDS) and administered a bio-behavioural survey. RDS is a peer-driven chain referral sampling technique designed for use among hard-to-reach populations and uses statistical adjustment to control for inherent biases introduced by the method’s non-random nature[27]. To initiate the chain referral process, ‘seeds’ were identified through contact with local organisations serving FSW. Seeds were well-connected members of the FSW community willing to recruit others in their social network. Three seeds were selected to begin the referral process, with another eleven added as accrual slowed. Each seed and each subsequent participant received three coupons to distribute to eligible members of their social network. Each coupon had an identifying code so that the recruitment chains could be traced, as well as an expiration date to control recruitment pace. Participants were reimbursed for their time and for travel costs upon completion of the survey and were additionally rewarded for every eligible participant that they recruited to the study. Recruitment continued until the target sample size was met.Recruitment continued until the target sample size was met. Sample size was calculated as the number needed to detect differences (odds ratio of 2.0) in HIV prevalence between participants with higher HIV-related protective behaviours with 95% confidence and 80% power.

Women aged 16 years or older who had exchanged sex for money, favours or goods in the past year and who presented a valid recruitment coupon were eligible for the study. Participants completed a structured survey via face-to-face interview in SiSwati or English with whichever one of four interviewers (two male, two female) was available at the time. All interviews took place in private at a study clinic in Manzini; which is the most populous Swazi city and located in the middle of the country. The questionnaire covered demographic characteristics, sexual behaviour, violence, substance use, discrimination, social capital and sexual health knowledge (S1 Text). Sexual behaviour questions included items on consistency in condom use separately for AI and VI in the past month with new clients, regular clients and non-paying partners, and condom use at last sex (VI or AI) with any partner type. The questionnaire did not include questions on the number of AI or VI sex acts. Participants were asked to report the size of their social network, defined as the number of other FSW the participant personally knows and has seen or talked to in the past six months, in order to account for bias introduced through the increased probability of recruiting FSW with comparatively larger networks. Additionally, participants were tested for HIV (using Unigold by Trinity Biotech and Determine HIV by Alere, with indeterminate samples sent to a laboratory for further testing) and syphilis (using Determine Syphilis by Alere) and referred for treatment if positive.

### Data analysis

Sample characteristics are presented as both crude and RDS-adjusted estimates. Adjusted estimates take into account participants’ varying network sizes. RDS-II weights were used and 95% confidence intervals (95%CI) were calculated by clustering the standard errors at the recruiter level[28].

We produced both crude and RDS-adjusted prevalence estimates of AI and AI with inconsistent condom use with 1)any partner type (i.e. with one or more partner type) and 2)by partner type among FSW reporting sex with that partner type. We derived inconsistent condom use during VI in the past month by partner type as well as the subsets who report 1) practicing VI only and 2) practicing AI and VI among FSW reporting that partner type. AI practice with inconsistent condom use was defined as reporting AI practice and using condoms most of the time, sometimes, rarely or never during AI in the past month, with the equivalent definition for VI with inconsistent condom use. We reported the proportion reporting a condom breaking or slipping during VI and during AI by partner type in the past month.

Interviewers’ characteristics or behaviour can influence how respondents answer questions, particularly of stigmatised topics like AI[29]. We therefore explored possible interviewer effects by calculating the intraclass correlation coefficient, which measures the percentage of total variance for a particular question that is attributable to the interviewer[30].

We examined the correlates of practice of AI and AI with inconsistent condom use using univariate and multivariable logistic regression models. We used Generalised Estimating Equations to account for clustering of participants by recruiter in the regression models[31] using a an exchangeable working correlation structure. Continuous variables were dichotomised at the median. Based on our review of the literature, we developed a conceptual framework of the dyadic, individual, community, and work environment and societal correlates of AI practice (S1 Fig). This framework was used to select potential variables for inclusion in the regression models. Some variables of interest (binge drinking, social participation and ability to negotiate condoms) were not included because they are believed to be of limited accuracy (e.g. several participants’ answers to the two drinking questions: ‘have you drunk in the past week’, and ‘number of drinks in the past week’ were contradictory). For variables which measured similar constructs (e.g. having been harassed, beaten or tortured), the variable with fewer missing cases was entered.

Personal characteristic variables included in the models were age, highest level of education (primary or lower/some secondary or higher), location having grown-up (urban/rural/foreign country), and number of dependents supported through sex work (0-2/3+). Included behavioural variables were number of sex acts per week (<5/5+), condom use at last sex with new or regular client (yes/no), number of new clients, of regular clients and of non-paying partners in the past month (each dichotomised at median), and any drug use in the past year (no/yes). Included social discrimination and violence variables included were ever having been blackmailed (no/yes), ever having been physically or verbally harassed (no/yes), ever having been raped since age 18 (no/yes), ever having felt afraid to access health services (no/yes), ever having felt afraid to walk in public please (no/yes) and a social cohesion score as a measure of social capital (detailed in S1 Table footnote). Included variables related to knowledge and access to information and services were knowing that AI conferred the highest sexual transmission risk (yes/no), having been tested for STIs in the past year (yes/no) and having received information on HIV prevention in the past year (yes/no). To control for the potential confounding of interviewer effects, we entered the respondents’ interviewer identification as dummy variables into the multivariable analysis.

Eleven of the 20 variables in the final AI model and ten of 19 in the AI with inconsistent condom use model contained missing data. In this context, a complete case analysis would have dropped 22% (n = 70) of the sample from the analysis. Missing values were therefore dealt with using multiple imputation chained equations, an iterative process that imputes multiple variables through posterior prediction distribution using a series of univariate chained equations[32]. We used ten iterations and combined the multiple datasets produced following Rubin’s rules[33]. Missing values for the outcome variables (AI and AI with inconsistent condom use practice) were not imputed, but were included in the imputation models as predictors[34,35].

The logistic models did not include RDS survey weights, as this is often unwarranted in regression modelling[36]. We conducted all analysis in R version 3.2.0[37] using the *RDS*[38], *geepack*[39], to fit the regressions and *mi*[40] and *mitml*[41] to conduct multiple imputation.

### Ethics

All participants provided written informed consent. Ethical approval was granted by review boards at the Swazi Ministry of Health, Johns Hopkins School of Public Health and Imperial College London.

## Results

### Survey participants

Ten of the 14 seeds recruited peers over a maximum of seven waves, resulting in a sample size of 325 women (S2 Fig). Sample characteristics are presented in Table 1 with both crude and RDS-adjusted estimates. The mean (median) age of the sample was 26 [25] years (range: 16–49). Most participants (74%) initiated sex work after reaching 18 years of age and had at least some secondary education (67%). Nearly half of the sample were living in Manzini (49%) at the time of the study, which is the most populous region of the country and where the study centre was located. The sample was equally split between having grown up in urban or rural areas. A large majority had never been married (96%) but most had at least one child (76%) and half financially supported three or more people through their sex work (52%). The most common primary place of work was in private homes (60%). Most FSW had no pimp (69%) and no other source of income beside sex work (67%). Just over half of the sample took only cash as payment (51%), with others also accepting goods. HIV prevalence was very high in the sample at 70%, while 8% tested positive for syphilis. Only 10% knew that AI carries the highest sexual HIV risk. Few women reported any lubricant use (21%), and of those, less than a third used condom-compatible lubricant. Crude and RDS-adjusted estimates of sample characteristics were largely similar. thThe intraclass correlation coefficient was high for the practice of AI and AI with inconsistent condom use, at 0.10 and 0.14, respectively, indicating that responses varied substantially by interviewer. Values for other variables were lower, ranging from 0.0–0.07(S1 Table).

**Table 1:** Selected characteristics of surveyed female sex workers in eSwatini in 2011(N = 325).

			Crude estimates		RDS-adjusted	
Variable	Categories	n	%	95% CI	%	95% CI
Age in years	≤20	64	20	14–26	30	19–41
	21–25	103	32	26–38	27	19–36
	26–30	84	26	20–32	26	17–35
	31+	74	23	17–29	17	10–25
Age started to sell sex	<18 years	83	26	20–32	31	20–41
(4 missing values)	18–21	121	38	32–44	39	28–49
	22+	117	36	31–42	30	21–39
Highest level of education	Primary or lower	106	33	28–38	33	24–42
	Secondary or higher	219	67	62–73	67	58–77
Region of residence	Manzini	159	49	43–55	51	40–61
	Hhohho	102	31	26–37	27	18–36
	Shiselweni	57	18	12–23	20	12–29
	Lubombo	6	2	0–8	2	0–7
	Outside eSwatini	1	0	0–6	0	0–0
Place grew up (3 missing values)	Urban	157	49	43–54	43	33–54
	Rural	153	48	42–53	52	41–62
	Foreign country	12	4	0–9	5	0–10
Marital status	Single or widowed	308	96	94–98	95	87–100
(4 missing values)	Married or cohabiting	13	4	2–6	5	0–13
Number of living children (1 missing value)	0	79	24	19–30	29	19–40
	1 or 2	182	56	51–62	55	44–66
	3+	63	19	14–25	15	9–22
Number of dependents	0–2	156	48	42–54	54	44–65
	3–5	114	35	30–41	33	23–43
	6+	55	17	11–23	13	7–19
Most common location for sex with clients (2 missing values)	Private home	195	60	55–66	61	51–72
	Hotel	87	27	22–32	27	17–38
	Car, street or park	33	10	5–16	9	5–13
	Bar/club or other	8	3	0–8	2	1–3
Has pimp	Yes	97	31	31–36	28	19–37
(9 missing values)	No	219	69	64–74	72	63–81
Income other than sex work (1 missing value)	Yes	108	33	28–39	29	20–39
	No	216	67	62–72	71	61–80
Payment type	Cash only	164	51	46–60	51	41–62
(5 missing values)	Cash and/or goods	156	49	43–54	49	38–59
HIV infected	Yes	223	70	66–76	62	51–73
(8 missing values)	No	94	30	25–35	38	27–49
Syphilis infected	Yes	24	8	5–10	9	2–17
(6 missing values)	No	295	92	90–95	91	8
Know type of sex with highest transmission risk (1 missing value)	Yes	34	10	7–14	8	4–12
	No	290	90	86–93	92	89–96
Any lubricant use with any partner, generally^†^ (4 missing values)	Yes	70	22	17–26	21	16–26
	No	251	78	74–83	79	74–85
AI practice with any partner in past month (5 missing values)	Yes	129	40	35–46	44	34–54
	No	191	60	54–65	56	46–66
AI with inconsistent condom use with any partner in past month (7 missing values)	Yes	104	33	28–38	34	26–42
	No	214	67	62–73	66	56–76

95% CI = 95% confidence interval. RDS-II method is used to calculate RDS adjustments.

^†^Question was: ‘Do you use lubricants?’

### Prevalence of anal intercourse and condom use during AI and VI

The prevalence of AI and AI with inconsistent condom use (RDS-adjusted) with any partner in the past month was 44% (95%CI:34–54%) and 34% (95%CI:26–42%), respectively (Table 1). The reported prevalence of AI and AI with inconsistent condom use ranged from 23% to 61% and 15% to 57% across interviewers, respectively. The two highest and two lowest AI prevalences were recorded by male and female interviewers, respectively (S1 Table).

AI prevalence did not vary by partner type, ranging from 36% (95%CI:27–44%) with non-paying partners to 39% (95%CI:30–48%) with regular clients (Table 2). The proportion reporting inconsistent condom use during AI, however, did vary by partner type; being most consistent with new clients and most consistent with non-paying partners. The same pattern was seen for inconsistent condom use during VI by partner type. The proportion reporting inconsistent condom use during AI was higher than during VI with each partner type, e.g.54% (95%CI:38–71%) reported inconsistent condom use during AI with new clients compared to 30% (95%CI:21–39%) during VI. A smaller proportion of FSW who exclusively practiced VI in the past month reported inconsistent condom use during VI with new and regular clients compared to FSW who practiced both VI and AI (Table 2). A higher proportion reported broken or slipped condoms during VI in the past month compared to during AI with both new and regular clients, but the proportions reporting broken condoms during AI and VI with non-paying partners were similar.

**Table 2:** Prevalence of anal and vaginal intercourse and inconsistent condom use during last month among Swazi female sex workers by partner type.

	Missing Values		Crude Estimates		RDS-Adjusted Estimates	
		n/N	(%)	(95% CI)	(%)	(95% CI)
With new clients (N = 297)						
Fraction reporting AI	3	100/294	34	29–40	37	29–46
Inconsistent condom use during AI	0	67/100	67	62–79	54	38–71
Inconsistent condom use during VI	2	75/297	25	20–30	30	21–39
Inconsistent condom use during VI, subset practicing VI only	0	39/197	20	14–25	27	15–38
Inconsistent condom use during VI, subset practicing VI and AI	2	35/98	36	26–45	35	21–50
Broken or slipped condom during AI	1	8/50	16	8–26	17	2–32
Broken or slipped condom during VI	8	81/288	28	23–34	26	17–34
With regular clients (N = 312)						
Fraction reporting AI	3	104/309	34	28–39	39	30–48
Inconsistent condom use during AI	0	77/104	74	65–82	69	53–86
Inconsistent condom use during VI	0	161/312	52	46–58	52	43–61
Inconsistent condom use during VI, subset practicing VI only	3	104/205	51	44–58	54	43–66
Inconsistent condom use during VI, subset practicing VI and AI	0	56/104	53	44–64	48	32–64
Broken or slipped condom during AI		12/46	26	15–39	28	11–45
Broken or slipped condom during VI	2	110/288	38	33–44	32	23–41
With non-paying partners (N = 284)						
Fraction reporting AI	1	93/283	33	28–39	36	37–44
Inconsistent condom use during AI	0	74/93	80	72–87	76	63–88
Inconsistent condom use during VI	0	189/284	67	61–72	62	53–71
Inconsistent condom use during VI, subset practicing VI only	1	133/190	70	63–76	69	59–80
Inconsistent condom use during VI, subset practicing VI and AI	0	55/93	59	49–69	53	36–65
Broken or slipped condom during AI	4	6/24	25	10–47	39	14–66
Broken or slipped condom during VI	27	84/206	41	34–48	37	26–47

AI = anal intercourse, VI = vaginal intercourse. Inconsistent condom use was defined as reporting using condoms with particular partner type ‘most of the time’, ‘sometimes’, ‘rarely’, and ‘never’ during anal or vaginal intercourse, as relevant. The denominator for the proportion practicing inconsistent condom use during AI is the number reporting AI, and the equivalent denominator is used for VI. If participants reported any condom use they were asked if any condoms during the past month had broken or slipped, the denominator in this case is those who reported any condom use (i.e. excluding those who report ‘never’ using condoms with that partner type). All those who reported AI with a particular partner type also reported VI with that partner type.

### Correlates of AI

Odds ratios measuring the association between AI and demographic, behavioural and structural factors are presented in Table 3. In univariate analysis, FSW reporting fewer sex acts in the past week, fewer new clients in the past month, never having been blackmailed and not feeling afraid to walk in public places because of being a sex worker were more likely to report AI practice. After adjustment for potential confounders, the multivariable regression results show that AI practice was more common among FSW who have at least some secondary education (adjusted Odds Ratio (aOR) = 1.92; 95%CI:1.03–3.57) and had grown up in rural areas (aOR = 1.90; 95%CI:1.09–3.32). FSW whose last sex act with a client was condomless were more likely to report AI (aOR = 2.09; 95%CI:1.07–4.08). FSW who had five or more new clients in the past month had 66% lower odds of practising AI (aOR = 0.33; 95%CI:0.16–0.68). The odds of reporting AI practice were halved among FSW who had ever been blackmailed (aOR = 0.50; 95%CI:0.25–0.98) and FSW who ever felt afraid to walk in public places (aOR = 0.46; 95%CI:0.25–0.87). Conversely, FSW who had been verbally or physically harassed because of being a sex worker (aOR = 2.09; 95%CI:1.16–3.74) or had been raped (aOR = 1.95; 95%CI:1.05–3.65) had around twice the odds of reporting AI practice. Correlates of AI with inconsistent condom use were similar to AI practice, with the exception that the aOR for having been blackmailed was closer to the null and had a wider confidence interval (S2 Table).

**Table 3:** Demographic, behavioural and structural correlates of anal intercourse in the past month with any partner, among Swazi female sex workers (stratified by AI practice, and univariate and multivariable logistic regression with clustered standard errors). Stratified analysis shows crude data, logistic regression results are from models with imputed missing data.

			AI practice/past month		No AI practice/past month		Univariate		Multivariable^†^	
Variable	Category	N	n	%	n	%	OR	95% CI	aOR	95% CI
**Personal characteristics**	** **	** **	** **	** **	** **	** **	** **	** **	** **	** **
Age	<26 years	167	69	54	98	51	Ref	-	Ref	-
	26+	153	60	47	93	49	0.88	0.56–1.36	1.04	0.59–1.84
Highest level of education	Primary or lower	104	35	27	69	36	Ref	-	Ref	-
	Some secondary or higher	216	94	73	122	64	1.54	0.90–2.62	1.92*	1.03–3.57
Grew up	Urban	157	60	47	97	51	Ref	-	Ref	-
	Rural	148	64	50	84	44	1.32	0.83–2.10	1.90*	1.09–3.32
	Foreign country	12	5	4	7	4	1.16	0.40–3.42	3.19	0.93–10.75
Number of dependents supported by sex work	0–2	153	60	47	93	51	Ref	-	Ref	-
	3+	167	69	53	98	49	1.09	0.71–1.67	1.10	0.66–1.83
**Individual behaviour**										
Number of sex acts/week^**‡**^	<5	162	80	64	82	44	Ref	-	Ref	-
	5+	152	46	37	106	56	0.45**	0.28–0.73	0.75	0.42–1.34
Condom use at last sex with new or regular client	Condom used	242	89	71	153	80	Ref	-	Ref	-
	Condomless	75	37	29	38	20	1.50	0.85–2.66	2.09*	1.07–4.08
Number of new clients/month (14 NAs)	<5	183	90	76	93	50	Ref	-	-	-
	5+	123	29	24	94	50	0.35***	0.21–0.58	0.33***	0.16–0.68
Number of regular clients/month	<7	184	78	62	106	56	Ref	-	-	-
	7+	131	48	38	83	44	0.83	0.54–1.28	1.40	0.78–2.49
Number of non-paying partners/month	0 or 1	206	77	60	129	68	Ref	-	Ref	-
	2+	113	51	40	62	33	1.41	0.89–2.22	1.18	0.67–2.06
Any drug use/year	No	207	82	65	125	66	Ref	-		
	Yes	108	45	35	63	34	1.08	0.67–1.72	1.00	0.57–1.74
**Social discrimination and violence **										
Ever blackmailed	No	210	95	74	115	60	Ref	-	Ref	-
	Yes	110	34	26	76	40	0.56*	0.33–0.95	0.50*	0.25–0.98
Ever physically or verbally harassed	No	125	49	38	76	40	Ref	-	Ref	-
	Yes	195	80	62	115	60	1.08	0.69–1.68	2.09**	1.16–3.74
Ever raped since age 18	No	180	63	53	117	67	Referent	-	Ref	-
	Yes	123	57	48	66	36	1.62	0.98–2.69	1.95*	1.05–3.65
Ever afraid to access health services	No	180	68	53	112	59	Referent	-	Ref	-
	Yes	140	61	47	79	41	1.27	0.81–2.00	1.54	0.86–2.78
Ever afraid to walk in public places	No	167	79	61	88	46	Referent	-	Ref	-
	Yes	153	50	39	103	54	0.54**	0.36–0.82	0.46*	0.25–0.87
Social cohesion score^**§**^	High	157	58	49	83	46	Ref	-	Ref	-
	Low	141	60	51	97	54	0.91	0.59–1.39	0.85	0.50–1.45
**Knowledge, information and services access **										
Knowledge of type of sex with highest transmission risk	Anal	34	15	12	19	10	Ref	-	Ref	-
	Other	286	114	88	172	90	0.84	0.42–1.66	0.79	0.32–1.98
Tested for STIs/year	Yes	232	36	28	52	27	Ref		Ref	** -**
	No	82	93	72	139	73	0.92	0.56–1.49	1.29	0.71–2.35
Received information on HIV prevention/year	Yes	272	109	85	163	86	Ref	-	Ref	-
	No	45	19	15	26	14	1.11	0.56–2.20	1.34	0.57–3.15

AI = anal intercourse, aOR = adjusted odds ratio, OR = odds ratio, STI = sexually transmitted infection, 95%CI = 95% confidence interval, Ref = reference level. *p<0.05, **p<0.01, ***p<0.001.

^†^ Multivariable results are mutually adjusted for all variables listed in this table. In addition to the variables listed, interviewer was entered into the model as a dummy variable in order to control for its potential confounding effect.

^‡^Condom use at most recent sex with new or regular clients was derived from two questions on condom use at last sex with new and regular clients separately

^§^ Social cohesion is an index comprised of a series of questions on relationship with other FSW. For more information, see S1 Table footnotes.

### Associations between anal intercourse, HIV and syphilis

Practice of AI and AI with inconsistent condom use was positively associated with testing positive for syphilis (aOR for syphilis infection among those practicing AI = 0.44; 95%CI:0.05–0.74) but had no association with HIV status (Table 4).

**Table 4:** Association between the practice of anal intercourse and anal intercourse with inconsistent condom use and HIV and syphilis infection.

Outcome	AI practice in past month					AI with inconsistent condom use in past month				
		Univariate		Multivariable^†^			Univariate		Multivariable^†^	
	n/N	OR	95% CI	aOR	95% CI	n/N	OR	95% CI	aOR	95% CI
Tested positive for HIV (8 missing values)	219/313	0.97	0.58–1.60	0.88	0.50–1.52	218/311	1.09	0.65–1.85	0.91	0.51–1.64
Tested positive for syphilis (6 missing values)	23/315	0.29*	0.08–0.79	0.24	0.05–0.74	22/313	0.30*	0.07–0.91	0.31	0.07–0.98

AI = anal intercourse, aOR = adjusted odds ratio, NA = number of missing values, OR = odds ratio, STI = sexually transmitted infection, 95%CI = 95% confidence interval, Ref = reference level. *p<0.05, **p<0.01, ***p<0.001.

^†^Multivariable models are adjusted for covariates that have previously been found to be significantly associated with HIV infection in this sample[3]: age, highest level of education, reporting STI symptoms in the past 12 months, reporting ever disclosing sex work to a health care worker and condom use during vaginal intercourse with new clients in the past month. These same covariates with the addition of the number of new clients in the past month were used to adjust the syphilis model.

## Discussion

AI practice in the past month was very common among this sample of Swazi FSW (RDS estimate = 44%) and a third reported AI with inconsistent condom use. While there are no other data on AI among Swazi FSW with which to compare our results, these estimates are similar to estimates from FSW in neighbouring KwaZulu-Natal in South Africa (43%[16] and 40%[42] reporting practicing AI as part of their service). Consistent condom use was lower during AI than during VI with each partner type. A third of the total sample reported AI with inconsistent condom use in the past month which, given the increased HIV transmission risk during AI, may substantially contribute to this population’s very high HIV prevalence, although no association was found between recent AI practice and HIV infection. Reporting any broken condoms in the past month was more common during VI than AI, but lack of data on the number of each type of sex act hinders the interpretation of this finding, as the total number of VI acts is likely to be higher than the number of AI acts.

Our results suggest that FSW who practice AI have fewer new clients and tend to have fewer sex acts. Several other studies have found that FSW typically charge more for AI than for VI[10,13,17], and practice it because of this financial incentive[43,44], so it is possible that those who practice AI do so in order to maximize sex-work revenue while reducing their number of clients. We have data on price per condom protected and condomless VI act in this sample, but not for AI acts. The reported mean fee for condomless VI (US$17) was over twice that for condom protected VI (US$8), and those practising AI were more than twice as likely to report that their last sex act with clients was condomless. This may imply that the same FSW are motivated by the financial incentive to practise both condomless VI and AI.

Our finding that those who report being verbally or physically harassed or having been raped are more likely to report AI is in agreement with other studies’ findings that victims of violence are more likely to practise AI[17,19–22]. However, we also found that those who report AI were less likely to be afraid to walk in public and less likely to have been blackmailed. This mixed picture may reflect AI being practised by two distinct groups of Swazi FSW, as described by qualitative researchers: one who felt that poverty left little choice other than to enter sex work, and the other who appreciate s the autonomy that the relatively lucrative work provides[23,24].

Despite a well-recognized heightened risk of transmission during condomless AI[5], we found no association between AI practice and HIV infection and an inverse association with syphilis infection in this cross-sectional sample. AI practice was measured over short time-periods (past month) which may not reflect this behaviour at the time of infection. A recent review also found that associations between AI and HIV prevalence were inconsistent in cross-sectional samples[6]. Prospective studies are more appropriate to determine causality and there is indeed strong evidence that AI enhances HIV risk in women[5].The transmission risk of syphilis during AI is less well understood, but is believed to likely be higher than during VI[45]. Our finding that the small number infected with syphilis are less likely to practice AI is therefore surprising and may be a result of residual confounding.

Foremost among this study’s limitations is the use of face-to-face interviews. Heterosexual AI is highly stigmatised in Southern Africa[46,47], and use of non-confidential interview methods is likely to have resulted in underreporting of AI and other sensitive topics included in the analysis[48–50]. AI reporting was shaped by substantial interviewer effects and we therefore adjusted for interviewer in the multivariable analyses. Reporting AI practice was more common with the male interviewers, but with only four interviewers we cannot conclude on the potential effect of interviewers’ gender. If interviewer gender does have an effect however, one reason may be that given the high demand for AI from their male clients, FSW may feel less shame in reporting AI practice to men as in their experience men are accepting of AI. Although this is an interesting question, we recommend that rather than conducting research to identify causes of interviewer effects, similar surveys in the future simply employ more confidential interview methods to collect data on AI practice and other stigmatised behaviours. We could not use the available data on lubricant use to explore the reasons for condom breakage as the recall periods differed, and while condom breakage was reported by partner type and type of sex act, lubricant use was not A further limitation is that the survey did not include questions on the number of AI and VI sex acts, without which it is not possible to estimate the contribution of AI practice to HIV transmission among Swazi FSW and to the wider Swazi epidemic. Additionally, while FSW from all regions of eSwatini were present in the sample, regions further away from the study centre were underrepresented and therefore the sample is not representative of FSW throughout the country. It is a strength that the questionnaire included several questions on violence and discrimination, however, the Lack of sex act data is a common weakness of behavioural surveys, with a systematic review of heterosexual AI practice among South Africans[51] identifying only one study which reported on frequency of AI acts among FSW, which found that around 20% of all sex acts were anal[52]. A recent study among Côte d’Ivoire FSW found that a similar proportion of sex acts were anal (21%) among the fifth of the sample who reported AI and mathematical modelling of these data suggest that 22% of new HIV infections could have been averted in this population had AI been substituted for VI [13]. If AI is practiced as frequently among Swazi FSW, then AI’s contribution to the country’s HIV epidemic is likely substantial[53].

There are a number of possible approaches to reducing the HIV transmission risk from AI among Swazi FSW. Tenofovir, the active component in oral pre-exposure prophylaxis (PrEP) has been found at higher concentration in rectal than vaginal tissue, and is likely more protective during receptive AI than VI[54–58]. Increasing access to PrEP could be effective for some FSW, although during a demonstration project adherence among FSW has been found to be low in neighbouring South Africa[59]. In the future, rectal microbicides or dual vaginal and rectal microbicides may also provide an option for FSW to protect themselves during AI[60]. However, given ease of access, interventions to increase condom use along with condom compatible lubricant in this population is likely to remain an efficient and cost-effective approach that cannot be overlooked. Counselling on proper condom and lubricant use may decrease the rate of condom breakage[61]. Additionally, decriminalisation of sex work as well as interventions to reduce violence victimisation may help reduce many of the structural barriers to safe sex practice faced by FSW[62].

## Conclusion

In conclusion, we found that AI is very commonly practised among Swazi FSW with all types of sex partners. Both condom use during AI and knowledge of HIV risk associated with AI is low. Taken together, these results suggest the importance of biomedical interventions that address HIV acquisition risks associated with anal intercourse combined with integration of education regarding safe anal sex in sexual health education programs in eSwatini.

## Supporting information

S1 Text Survey questionnaire in English and SiSwathi. (DOCX)

S1 Table Characteristics and behaviours included in the multivariable analysis, stratified by interviewer. (DOCX)

S2 Table Demographic, behavioural and structural determinants of practicing anal intercourse with inconsistent condom use in the past month with any partner, among the whole sample of Swazi female sex workers. (DOCX)

S1 Fig A conceptual framework of anal intercourse practice among female sex workers. (DOCX)

S2 Fig RDS recruitment network tree. (DOCX)

## List of abbreviations

AI: Anal intercourse
VI: Vaginal intercourse
FSW: Female sex workers
PrEP: Pre-exposure prophylaxis
RDS: Respondent-driven sampling
OR: Odds ratio
aOR: Adjusted odds ratio
95% CI: 95% confidence interval
