# Correction: Prevalence and correlates of anal intercourse among female sex workers in eSwatini

**DOI:** 10.1371/journal.pone.0231487

**Published:** 2020-04-14

**Authors:** Branwen N. Owen, Mathieu Maheu-Giroux, Sindy Matse, Zandile Mnisi, Stefan Baral, Sosthenes C. Ketende, Rebecca F. Baggaley, Marie-Claude Boily

The second author's name appears incorrectly. The correct name is: Mathieu Maheu-Giroux. The correct citation is: Owen BN, Maheu-Giroux M, Matse S, Mnisi Z, Baral S, Ketende SC, et al. (2020) Prevalence and correlates of anal intercourse among female sex workers in eSwatini. PLoS ONE 15(2): e0228849. https://doi.org/10.1371/journal.pone.0228849
