# Supplementary material for: Prevalence and correlates of anal intercourse among female sex workers in eSwatini
Source: PLoS One. 2020 Feb 11;15(2):e0228849. doi: 10.1371/journal.pone.0228849 (PMC7012411; doi:10.1371/journal.pone.0228849)
Supplement: S1 Text — (DOCX) [file pone.0228849.s001.docx]

**Swaziland Commerical Sex Worker Survey Instrument**

**2011**

**Swaziland Commerical Sex Worker: Consent Form**

**Introduction*/Singeniso***

This consent form explains the research study you are being asked to join. I will read and review this form with you now and give you an opportunity to ask any questions about the study before you agree to join the study. You may also ask questions at any time after you join the study.

*Lelifomu lekuvumelana lichaza kabanti ngalolucwaningo locelwa kutsi ube yincenye yalo.Ngitalifundza lelifomu embikwakho bese ngikunika litfuba lekutsi ubute noma ngabe ngumuphi umbuto longaba nawo mayelana nalolucwaningo ungakatsatsi sinyatselo sekuba yincenye yalo.Uvumelekile kubuta noma ngabe ngumuphi umbuto lusachubeka lolucwaningo.*

**Purpose/*Injongo***

You are invited to take part in a research study. The purpose of this study is to learn about the experiences of sex workers, and improve services for sex workers. To join the study is voluntary. You may refuse to join, or you may withdraw your consent to be in the study, for any reason, without penalty.

*Umenywa kutsi ube yincenye yelucwaningo. Injongo yalolucwaningo kwati kabanti ngetimo lababhekana nato labatfola inzuzo ngekulala, bese setamala kutfutfukisa indlela labangasitakala ngayo kuleto timo. Kungenela lolucwaningo kukutikhetsela kwakho.Ungakhetsa kungalungeneli noma ukhetse kutsi ungabe usachubeka nalo noma ngabe ngutiphi tizatfu, ngaphandle kwekujeziswa.*

**Why you are being asked to participate**

***Ucelwe leni kutsi ube yincenye***

You are being invited to participate in this study because you reported exchanging sex for money in the last 12 months.

*Ucelwa kutsi ube yincenye yalolucwaningo ngoba uvumile kutsi uke watfola imali ngekulala etinyangeni letilishumi natimbili letendlulile.*

**Procedures/*Indlela yekusebenta***

We are asking individuals who identify as sex workers to participate in a survey, HIV test and syphilis test. This includes participating in an HIV and Syphilis rapid test which includes pre- and optional post-test counseling. If found positive for HIV, we will provide counseling and referrals for treatment.If found positive for Syphilis, we will provide counseling and treatment for this infection. We will conduct one face-to-face survey with you. The survey would take place in a private room. We expect that the survey will take approximately 60 minutes of your time. Also, the rapid tests for HIV and Syphilis will take an estimated 20 additional minutes. We estimate that study participation will take approximately 90 minutes plus travel to the study site. If you choose to recruit others, we expect that it will take approximately 5 minutes to inform your contact about the study as well as the travel time needed to meet your colleague.

In the survey, we will ask you questions about your background, attitudes toward HIV, behaviors, and past experiences related to sex workand HIV. You do not have to answer any question that you feel uncomfortable with.Though testing for HIV and syphilis is a component of the research, it is your choice to receive counseling and/or to know your results.

After completing the research, we will also ask you to recruit three additional individuals who identify as sex workers. You will be asked to return two weeks after your initial visit in order to receive compensation based on the number of individuals that you recruited who presented to participate in the study.

*Sicela labo labatfola inzuzo ngekulala kutsi babe yincenye yalolucwaningo, bese bapotjolwa ingati kubona ligciwane le-HIV kanye nabogcunsula.Loku kufaka ekhatsi kwelulekwa ungakacali kubotjolwa uphindze welulekwe nasewucedzile kupotjolwa.Nangabe utfolakale kutsi unalo leligciwane lembulalave, utakwelulekwa ngalokufanele uphindze uchunyaniswe nalapho ungatfola khona kwelashwa.Nawutfolakala unagcunsula, utawelulekwa ngalokufanele uphindze welashwe.Kunemibuto lesitakubuta yona buso nebuso.Itawubutwa endzaweni lephephila.Lemibuto ibhekeke kutsi itsatse sikhatsi lesingaba li-awa.Lokuhlolwa ligciwane le-HIV kanye nabogcunsula kona kutawutsatsa imizuzu lengaba ngemashumi lamabili.Singabekisa sitsi lolucwaningo selulonkhe lutawutsatsa imizuzu lengaba ngu-90 ngaphandle kwekuhamba nawuseta kutowungenela lolucwaningo.Kungenteka kutsi utsandze nekutsi ugcugcutele labanye kutsi babe yincenye yalolucwaningo.Sicabanga kutsi kutawuba yimizuzu lesihlanu kubachazela ngalolucwaningo.*

*Kulolucwaningo sitakubuta imibuto ngelimuva lakho, indlela locabanga ngayo (noma lotsatsa) ngayo nge-HIV, indlela lotiphatsa ngayo, netintfo letentekile emuva letiphatselene nemsebenti wekutfola inzuzo ngekulala kanye ne-HIV.Awukabhekeki kutsi uphendvule nangabe umbuto ungakhululeki ngekuwuphendvula. Noma kupopola ligciwane le HIV kanye nagcunsula (syphilis) kuyincenye lebalulekile yalolucwaningo, kukuwe kutsi utikhetsele kutsi uyafuna yini kwelulekwa uphindze utfole imiphumela yengati emva kwekupotjolwa.*

*Nasewulucedzile lolucwaningo, sitawuphindze sicele kutsi ukhulumisane nalabenye labatsatfu lobatiko kutsi bakuwo lomsebenti wekutfola inzuzo ngekulala kutsi nabo babe yincenye yalolucwaningo.Utawucelwa kutsi ubuye emuva kwemaviki lamabili kusukela manje utowutfola sibongo lesitobalwa ngebantfu lobamemile lakakhonile kutsi bagcine babe yincenye yalolucwaningo.*

**Risks/Discomforts *Bungoto/Kungakhululeki***

We do not think that being part of this study will create significant risks for you. In the survey I will be asking you questions about your day-to-day experience. Some of the topics may be emotional or difficult to discuss. You do not have to answer any questions that you would prefer not to answer. If you feel that you would like to speak to someone after the survey we can refer you to appropriate services.

There is a risk that if somebody finds out that you are participating in this study that they will discover that you are a sex worker. We will do everything we can to protect this information. Your name will not be collected at any point during this study.

Whether or not you decide to participate in the study will not affect your involvement in any other research study or any of the services you currently receive. You can stop the study at any time without penalty.

*Asicabangi kutsi kuba yincenye yalolucwaningo kungahle kukuletsele bungoti tsite. Kulolucwaningo ngitokubuta ngetintfo lohlangabetana nato onkhe malanga. Letinye taletihloko tingahle tikwente utsintseke emoyeni nome kube lukhunyana kutsi ukhulume nyato.Akukadzingeki kutsi uphendvule indzaba lotivela ungakhululeki kukhuluma ngawo.Nawuva kutsi udzinga kukhuluma nalomunye umuntfu emuva kwalolucwaningo, sitakulungisela labo labafanele.*

*Kukhona bungoti bekutsi nangabe kungaba khona lowatiko kutsi ube yincenye yalolucwaningo angati kutsi wena utfola inzuzo ngekulala. Sitakwenta konkhe lokusemandleni etfu kutsi kugcineke kuyimfihlo, uvikeleke. Ligama lakho angeke libe khona kunoma nguyiphi incenye yalolucwaningo.*

*Uma ngabe ukhetsa kulungenela noma kungalungeleli lolucwaningo, loku angeke kukuvimbele kutsi ungabe usalingenela lolunye lucwaningo, futsi angeke kuvimbe kutsi utfole lusito lolutfolako manje.Ungayekela kuchubeka nalolucwaningo noma ngabe ngunini ngaphandle kwesijeziso.*

**Benefits/*Inzuzo***

There is no direct benefit to you from participating in this study. However, we hope the findings will help create HIV prevention, care and treatmentprograms that better meet the needs of sex workers.

*Kute lusito lolutfolakala ngekuba yincenye yalolucwaningo.Kepha siyetsemba kutsi imiphumela yalo itawusita etinhlelweni tekuvikela, kunakekela nekwelapha ligciwane le-HIV kubantfu labatfola inzuzo ngekulala.*

**Reimbuisement**

You will receive a reimbursement for your travel and time after the completion of the first interview. We will also ask your help recruiting up to three additional participants. For each new person that you successfully recruit, you will receive E20 . When you return after two weeks to collect the recruitment reimbursement, you will also be given a travel reimbursement.

*Nawucedza lolucwaningo lwekucala, utawutfola imali llesincephetelo sekugibela nawuta lapha kanye nesikhatsi sakho losicitsitsile kulolucwaningo. Sitawucela nekutsi uphindze umeme nalabanye labatsatfu kutsi bangenele lolucwaningo.Utawutfola imali lengu E20.00 kuloyo naloyo lomumemile wagcina aphumelele kuta watoba yincenye yalolucwaningo.Utawubuya emvakwemaviki lamabili utolandza lesincephetelo sabo E20.00 salaba lobamemile.Nangaleso sikhatsi, utawuphindze uncephetelwe ngemali yekugibela.*

**Protecting data confidentiality/*Kuvikela imfihlo***

No identifying information will be collected. Survey data will be identified only by a unique ID number.

*Kute lokusho kutsi ungubani lokutoba yincenye yalolucwaningo.Kutawusentjetiswa tinombolo tsite nakusentjetwa kulolucwaningo*.

**Voluntariness/*Kutinikela***

Your participation in this research project is completely voluntary. You have the right to withdraw from the research study at any time. You should ask the local study coordinator, Zandile Mnisi if you have any questions about this research study. You may ask her questions in the future if you do not understand something that is being done.

*Kuba yincenye kwakho kulolucwaningo kukutinikela.Unelilungelo lekuyekela noma ngunini.Uma unemibuto ngalolucwaningo, ungatsintsa umchumanisi longu Zandile Mnisi.Ungambuta imibuto noma ngabe selwengcile lolucwaningo nangabe kukhona longakakucondzi.*

**Who do I call if I have questions or problems?**

***Ngishayela bani nanginemibuto noma tinkinga?***

- Call the local study coordinator, Zandile Mnisi, in Swaziland at 76036500, or the study Principal Investigator, Dr. Stefan Baral in the United States at 0014105028975
- If you have any questions about your rights as a research participant, or if you think you have not been treated fairly, you may call the Ministry of Health and Social Welfare at 404-2431.
- *Ungashayela umchumanisi walolucwaningo longu Zandile Mnisi ku 76036500 noma lophetse lolucwaningo Dr. Stefen Baral eMelika ku 0014105028975.*
- *Nangabe ufuna kubuta ngemalungelo akho njengemunftu loyincenye yalolucwaningo noma ngabe unesikhalo ngekungaphatseki kahle, ungashayela litiko letemphilo ku 404-2431.*

**Permission to proceed/*Imvume yekuchbeka***

Is it okay to proceed with the survey?

Is it okay to proceed with the HIV test and syphilis test?

*Singachubeka yini nalolucwaningo?*

*Singachubeka yini sihlole i-HIV kanye nabogcunsula?*

“I have read the consent form completely before the participant and he/she voluntarily agreed to participate in the study.”

*“Ngilifundzile lelifomu lekuvumelana ngalicedza embikwalona locelwe kuba yincenye yalolucwaningo kwaba nguye lotivumelako kulungenela.”*

Signature of Nurse

Date

Signature of Interviewer

Date

**Interviewer/Nurse:** Please keep one signed copy of the consent for the PI.

**Information Required for ALL INDIVIDUALS**

**Year**

**Month**

**Day**

**Interviewer ID**

**Unique ID (Place Label)**

**Personal Network Size (degree)**

**“How many different people do you know personally who are sex workers?”**

***“Bangakhi labanye lobatiko wena lebakulomsebenti wekutfola inzuzo ngekulala?”***

**“Of these, how many have actually you seen or talked to at least once in the last 6 months?”**

**“Kulaba lobatiko, bangakhi loke wababona noma wakhuluma nabo lokungenani kanye kuletinyanga letisitfupha letendlulile?”**

**ParticipantTicket Number**

**Ticket Numbers provided to participant for recruitment**

**Have you exchanged sex for money, favours or goods within the past 12 months?**

***Uke watfola yini imali, imphahla noma wentelwa lokutsite njengembadalo yekulala etinyangeni letingu 12 letendlulile?***

**Eligiblity Status**

1= Eligible (Continue with Survey)

2= Not Eligible (STOP HERE)

**Do you agree to participate in this study?**

***Uyavuma yini kuchubeka nalolucwaningo?***

**Eligiblity Status**

1= Eligible (Continue with Survey)

2= Not Eligible (STOP HERE)

If Consented, interviewer sign (Continue with Survey)

If Refused (STOP HERE)

**Consent Status from Verbal Consent Script**

**Module 1 – Demographic and Socio Economic Information**

“This first set of questions are about your background”

*“Lemibuto lesitawucala ngayo iphatselane nelimuva lakho”*

| **No.** | **Question** | **Coding** | **Response** |
| --- | --- | --- | --- |
| **1.1** | How old are you?  *Uneminyaka lemingakhi?* | **Age in Years**  88= No Response  99= Don’t Know | \|__\|__\| |
| **1.2** | What was your nationality at birth?  *Bebuyini buve bakho nawutalwa?* | 01= Swazi  02= Mozambique  03= South African  04=Other African  05=Other 🡪 _________________  88= No Response  99= Don’t know | \|__\|__\| |
| **1.3** | What is the highest level of your education?  *Ufundze wagcinaphi?* | 00= Never attended school  01= Some Primary School  02= Completed Primary  03= Some secondary or high School  04= Completed secondary or high school  05=post HS Vocational Training  06= post HS College/University  88= No Response  99= Don’t know | \|__\|__\| |
| **1.4** | What was your income last month (in EZ)?  *Kube ngumalini umholo wakho enyangeni leyendlulile (Emalangeni)?* | **Record number in box**  88= No Response  99= Don’t know | \|__\|__\|\|__\|__\| |
| **1.5** | In the last 12 months how many places have you lived?  *Etinyangeni letingu 12 letendlulile uke wahlala etindzaweni letingakhi?* | 00= No place  01= 1  02= 2-3  03= 4 or more  88= No Response  99= Don’t know | \|__\|__\| |
| **1.6** | How long have you lived in Swaziland?  *Sewunesikhatsi lesingakanani uhlala lapha kaNgwane?* | **All numbers  (Write response in months and years)**  88= No Response  99= Don’t know | \|__\|__\| - \|__\|__\|  mm yy |
| **1.7** | Where did you grow up?  *Ukhulelephi?* | 01= Urban  02= Rural  03=Foreign Country  88= No Response  99= Don’t know | \|__\|__\| |
| **1.8** | Which region do you currently stay?  *Uhlala kusiphi sifundza?* | 01= Hhohho  02= Manzini  03= Shiselweni  04= Lubombo  05= Outside Swaziland  88= No Response  99= Don’t Know | \|__\|__\| |
| **1.9** | What kind of place do you stay currently?  *Uhlala njani kulendzawo lokuyo?* | 00= No place to live / homeless **(Skip to 1.11)**  01= Renting place  02= Own place  03= Staying with someone  04= Other🡪 ________________  ___________________________  88= No Response  99= Don’t know | \|__\|__\| |
| **1.10** | In the place that you stay currently, who stays there with you?  **(Mark all that apply.)**  *Kulendzawo lohlala kuyo manje, uhlala nabani?*  *(Maka konkhe lokufanele)* | 00= No one/lives alone  01= Sexual partner  02= Relatives  03= Friend(s)  04= Colleague(s)  05= Other🡪________________  __________________________  88= No Response  99= Don’t know | \|__\|__\|  \|__\|__\|  \|__\|__\|  \|__\|__\| |
| **1.11** | Have you ever resided outside of Swaziland in the last 12 months?  *Uke wahlala yini ngaphandle kwaleli etinyangeni letingu 12 letendlulile?* | 00= No  01= Yes  88= No Response  99= Don’t know | \|__\|__\| |
| **1.12a** | What is your marital status?  *Umephi kutekutsatsana?* | 01= Married  02= Cohabitating  03= Divorced/Separated**(Skip to 1.13)**  03= Widowed**(Skip to 1.13)**  04= Single/Never Married**(Skip to 1.13)**  05= Other 🡪_____________ **(Skip to 1.13**  88= No Response  99= Don’t Know | \|__\|__\| |
|  | **1.12b** Does your husband or partner that you live with know that you sell sex?  *Iyati yini indvodza yakho noma singani sakho kutsi utfola inzuzo ngekulala.* | 00= No  01= Yes  88= No Response  99= Don’t know | \|__\|__\| |
| **1.13** | How many living children do you have?  *Bangakhi bantfwana bakho labaphilako?* | **All numbers (write 00 if no children)**  88= No Response  99= Don’t Know | \|__\|__\| |

**Module 2 – Human Rights and Exposure to Violations**

“Now we are going to ask you questions about how you have felt or been treated by other as a result of you selling sex. Please do not feel bad about answering as it will remain confidential”

*“Nyalo sesitakubuta imibuto ngendlela lewuke waphatfwa ngayo ngulabanye ngenca yekutfola imali ngekulala. Ungesabi kuphendvula ngoba sitakugcina loku kuyimfihlo.*

|  | **COMMUNITY/*UMANGO*** |  |  |
| --- | --- | --- | --- |
| **2.0** | Have you ever felt excluded from family gatherings as a result of you selling sex?  *Wake wativela yini umndeni wakho ungakumbandzakanyi etintfweni labatentako ngenca yekutsi utfola inzuzo ngekulala?* | 00= No  01= Yes  88= No Response  99= Don’t know | \|__\|__\| |
| **2.01** | Have you ever felt that family members have made discriminatory remarks or gossiped about you because of you selling sex?  *Bemndeni wakho bake bawasho yini emagama tsite ekukubandlulula noma bakuhleba ngenca yekutsi utfola inzuzo ngekulala?* | 00= No  01= Yes  88= No Response  99= Don’t know | \|__\|__\| |
| **2.02** | Have you ever felt rejected by your friends as a result of you selling sex?  *Wake wativela yini ujikelwa bangani bakho ngence yekutsi utfola inzuzo ngekulala?* | 00= No  01= Yes  88= No Response  99= Don’t know | \|__\|__\| |
| **2.03** | Have you ever felt rejected by other SEX WORKERS as a result of you selling sex?  *Wake wativela yini ujikelwa ngulabanye labatfola inzuzo ngekulala ngenca yekutsi nawe utfola inzuzo ngekulala?* | 00= No  01= Yes  88= No Response  99= Don’t know | \|__\|__\| |
| **2.04** | Have you ever lost employment as a result of you selling sex?  *Wake walahlekelwa yini ngumsebenti ngenca yekutsi uyatsengisa?* | 00= No  01= Yes  88= No Response  99= Don’t know | \|__\|__\| |
| **2.05** | Have you ever been denied educational opportunities, like access to school, as a result of you selling sex?  *Wake walelwa yini ematfuba ekufundza, njengesikolwa, ngenca yemsebenti yekutfola inzuzo ngekulala?* | 00= No  01= Yes  88= No Response  99= Don’t know | \|__\|__\| |
| **2.06** | Are there safe places in your area where you can go to socialize with other sex workers?  *Tikhona yini tindzawo letiphephile endzaweni yakini lapho ungakhonakuhlanganyela nekucocisana nalabanye labakulomsebenti?* | 00= No  01= Yes  88= No Response  99= Don’t know | \|__\|__\| |
|  | **HEALTH SYSTEM** |  |  |
| **2.07** | Have you ever felt afraid to seek health care services as a result of you selling sex?  *Wake kwenteka yini wesaba kufuna lusito lwetemphilo ngenca yekutsi utfola inzuzo ngekulala?* | 00= No  01= Yes  88= No Response  99= Don’t know | \|__\|__\| |
| **2.08** | Have you ever been denied health services as results of you selling sex?  *Wake walelwa yini kuniketwa lusito lwetemphilo ngenca yekutsi utfola inzuzo ngekulala?* | 00= No  01= Yes  88= No Response  99= Don’t know | \|__\|__\| |
| **2.09** | Have you ever felt that you have received lower quality of care within a health center as result of you selling sex?  *Wake wativela yini unganakeleleki kahle kubetemphilo ngenca yekutsi utfola inzuzo ngekulala?* | 00= No  01= Yes  88= No Response  99= Don’t know | \|__\|__\| |
| **2.10** | Have you ever been tested for HIV without consent as a result of your selling sex?  *Wake waphocelelwa yini kutsi uhlolwe ligciwane leHIV ungakavumi ngenca yekutsi utfola inzuzo ngekulala?* | 00= No  01= Yes  88= No Response  99= Don’t know | \|__\|__\| |
| **2.11** | Have you ever had difficulty in accessing health care services as a result of you selling sex?  *Wake waba nabo yini bulukhuni bekutfola lusito lwetemphilo ngenca yekutsi utfola inzuzo ngekulala?* | 00= No  01= Yes  88= No Response  99= Don’t know | \|__\|__\| |
| **2.12** | Have you ever heard health care providers gossiping about you as a result of you selling sex?  *Wake weva yini tisebenti tetemphilo tikuhleba ngekutsi utfola inzuzo ngekulala?* | 00= No  01= Yes  88= No Response  99= Don’t know | \|__\|__\| |
| **2.13** | Have you ever been tortured as a result of you selling sex?  *Wake wahlukubetwa yini ngekuviswa buhlungu ngenca yekutsi utfola inzuzo ngekulala?* | 00= No  01= Yes  88= No Response  99= Don’t know | \|__\|__\| |
| **2.14** | Have you ever felt any legal discrimination as a result of you selling sex?  *Wake wabandlululwa yini betemtsetfo ngenca yekutsi utfola inzuzo ngekulala?* | 00= No  01= Yes  88= No Response  99= Don’t know | \|__\|__\| |
|  | **SOCIETY** |  |  |
| **2.15** | Have you ever felt that the police refused protection as a result of you selling sex?  *Wake wativela yini ungavikelwa ngemaphoyisa ngesizatfu sekutsi utfola inzuzo ngekulala?* | 00= No  01= Yes  88= No Response  99= Don’t know | \|__\|__\| |
| **2.16** | Have you ever felt any verbal and physical harassment as a result of you selling sex?  *Wake wahlukubeteka yini ngekukhuluma noma kushaywa (noma kulinyatwa ngenca yekutsi utfola inzuzo ngekulala?* | 00= No  01= Yes  88= No Response  99= Don’t know | \|__\|__\| |
| **2.17** | Have you ever been blackmailed as a result of you selling sex?  *Wake wesatjiswa yini ngekutsi ukhokhe lokutsite kuze timfihlo takho tekutsengisa ngemtimba tingembulwa?* | 00= No  01= Yes  88= No Response  99= Don’t know | \|__\|__\| |
| **2.18** | Have you ever been arrested on false charges because of you selling sex?  *Wake waboshwa yini ngalokungasilo liciniso ngenca yekutsi utfola inzuzo ngekulala?* | 00= No  01= Yes  88= No Response  99= Don’t know | \|__\|__\| |
| **2.19** | Have you ever felt scared to walk around in public places as result of you selling sex?  *Wake wativela yini unekwesaba kuhamba emkhatsini walabanye bantfu ngenca yekutsi utfola inzuzo ngekulala?* | 00= No  01= Yes  88= No Response  99= Don’t know | \|__\|__\| |
| **2.20a** | Have you ever been beaten up as result of you selling sex?  *Wake washaywa ngenca yekutsi utfola inzuzo ngekulala?* | 00= No**(Skip to 2.21)**  01= Yes  88= No Response  99= Don’t know | \|__\|__\| |
|  | **2.20b** In the past year, how many times have you been beaten up as a result of you selling sex?  *Ushaywe kangaphi ngenca yekutsi utfola inzuzo ngekulala esikhatsini lesinganyetinyanga letingu 12 letendlulile?* | 01= 1-3  02= 4-6  03= 7-9  04= 10 or more  88= No Response  99= Don’t know | \|__\|__\| |
|  | **2.20c**Who was responsible for beating you up? **(Mark all that apply, ask about each)**  *Ngubani lona labekakushaya?*  ***(Maka konkhe lokufanele, futsi buta ngako konkhe)*** | 01=Uniformed officers (police, military, security etc)  02=Family member  03=Regular partner (not a client)  04=One-time client  05=Regular client  Regular partner, not a client  06=Your manager/pimp  88= No Response  99=Don’t Know | \|__\|__\| |
| **2.21** | In the time since you were 18 years old, have you ever been forced to have sex when you did not want to?  *Kusukela waba neminyaka lengu 18, uke waphocelelwa yini ngulomunye kutsi ulale ungafuni?* | 00= No **(Skip to 2.22a)**  01= Yes  88= No Response  99= Don’t know | \|__\|__\| |
|  | **2.21b** In the time since you were 18 years old, how many times have you been forced to have sex when you didn’t want to?  *Kusukela waba neminyanga lengu 18, kukangaphi lapho wake waphocelelwa nyulomunye kutsi ulale ungafuni?* | 01= 1-2  02=3-4  03=5-6  04= 6 or more  88= No Response  99= Don’t Know | \|__\|__\| |
|  | **2.21c**Who was responsible for forcing you to have sex when you did not want to? **(Mark all that apply, ask about each)**  *Ngubani lona labekuphocelela kutsi uye ecansini ungafuni?*  ***(Maka konkhe lokufanele, buta ngakunye)*** | 01=Uniformed officers (police, military, security etc)  02=Family member  03=Regular partner (not a client)  04=One-time client  05=Regular client  Regular partner, not a client  06=Your manager/pimp  88=No Response  99=Don’t Know | \|__\|__\| |
|  | **2.21d** Who did you tell about this/these incidents?  **(Multiple answers are acceptable)**  *Ngubani lowamtjela ngaloku lokwakwehlela?*  ***(Timphendvulo letinyenti temukelekile)*** | 00=Nobody **(Skip to 2.22a)**  01=Friend  02=Family Member  03=Police  04=Healthcare worker  05=Other ->  88=No Response  99=Don’t Know | \|__\|__\|  \|__\|__\|  \|__\|__\|  _____ |
|  | **2.21e** Was the person who forced you to have sex against your will ever legally prosecuted?  *Lomuntfu lowakuphocelela kulala naye wabhekana yini nemtsetfo?* | 00= No  01= Yes  88= No Response  99= Don’t know | \|__\|__\| |
| **2.22a** | Have you ever been to jail or prison?  *Wake wavalelwa yini waba sejele noma waba sesitokisini?* | 00= No **(Skip to Module 3)**  01= Yes  88= No Response  99= Don’t know | \|__\|__\| |
|  | **2.22b** Were you ever arrested because you were selling sex?  *Wake waboshelwa yini lomsebenti wekutfola inzuzo ngekulala?* | 00= No  01= Yes  88= No Response  99= Don’t know | \|__\|__\| |

**Module 3 – Personal History**

“Now we are going to ask you questions about your history with selling sex. Please do not feel bad about answering as it will remain confidential.”

*“Sesitawubuta imibuto mayelana nemlandvo wakho kulomsebenti wekutfola inzuzo ngekulala. Ngiyacela kutsi ukhululeke ungesabi ngoba konkhe lesitakukhuluma nawe kutawugcinwa kuyimfihlo.”*

| **No.** | **Question** | **Coding** | **Response** |
| --- | --- | --- | --- |
| **3.00** | How old were you the first time you traded or sold sex or sexual acts in exchange for money, favours or goods?*Wawunamingakhi iminyaka ngesikhatsi ucala kulala ngesizatfu sekutsi ufuna kutfola imali noma lenye inzuzo lephatsekako?* | **Record agein box**  88= No Response  99= Don’t know | \|__\|__\| |

|  | **3.00a** Who introduced you into selling sex?  *Ngubani lowakucalisa lomsebenti?* | 00= No one else was involved  01= Husband or boyfriend**(Skip to 3.01)**  02= Stranger**(Skip to 3.01)**  03= Friend **(Skip to 3.01)**  04= Family member**(Skip to 3.01)**  05= Other 🡪________  **(Skip to 3.01)**  88= No Response  99= Don’t know | \|__\|__\| |
| --- | --- | --- | --- |

|  | **3.00b**If decided on your own, what were the reasons for this decision?  *Nangabe kwaba sincumo lowatitsatsela sona, kwaba yini tizatfu taloko?* | 01= Only way to feed myself  02= Only way to feed my family  03= I did not know of any other way to earn money  04= I had to pay off a debt  05= Other 🡪88= No Response  99= Don’t know | \|__\|__\|  \|__\|__\| |
| --- | --- | --- | --- |
| **3.01** | How many people are financially supported by your selling sex, not including yourself?  *Bangakhi bantfu, ngaphandle kwakho, labasitakalako ngemali loyitfola kulomsebenti?* | **Record number in box**  88= No Response  99= Don’t know | \|__\|__\| |

| **3.02** | Have you told any member of your family that you sell sex?  *Kukhona yini lowake wamtjela emndenini kutsi utfola inzuzo ngekulala?* | 00= No  01= Yes  88= No Response  99= Don’t know | \|__\|__\| |
| --- | --- | --- | --- |
| **3.03** | Does anyone in your family know that you sell sex?  *Kukhona yini lowatiko emndenini wakho kutsi wena utfola inzuzo ngekulala?* | 00= No  01= Yes  88= No Response  99= Don’t know | \|__\|__\| |
| **3.04** | Have you told any health care worker that you sell sex?  *Kukhona yini sisebenti setemphilo lowake wasitjela kutsi wena utfola inzuzo ngekulala?* | 00= No  01= Yes  88= No Response  99= Don’t know | \|__\|__\| |
| **3.05** | On average, about how many clients do you have each week?  *Ngalokuvamile, mangakhi emakhasimende akho ngeliviki?* | **Record number in box**  88= No Response  99= Don’t know | \|__\|__\| |
| **3.06a** | On average, how many days per month do you sell sex?  *Ngalokuvamile, mangakhi emalanga enyangeni lapho khona ulala nemakhasimende?* | **Record number in box**  88= No Response  99= Don’t know | \|__\|__\| |

|  | **3.06b** On average, how much money do you make per month selling sex?  *Ngalokuvamile, utfola malini ngenyanga kulebhizinisi?* | **Record number in box**  88= No Response  99= Don’t know | \|__\|__\|\|__\|__\| |
| --- | --- | --- | --- |
| **3.07a** | Do you have another income aside from what you make in sex work?  *Ikhona yini lenye indlela yekungenisa imali ngaphandle kwekulala?* | 00= No **(Skip 3.08a)**  01= Yes  88= No Response  99= Don’t Know | \|__\|__\| |
|  | **3.07b** On average, how much money do you make per month from other income?  *Ngekubekisa kwakho, utfola malini nje ngenyanga kulolomunye umsebenti?* | **Record number in box**  88= No Response  99= Don’t know | \|__\|__\|\|__\|__\| |
| **3.08a** | In the past 12 months, in which region did you practice sex work the most?  *Etinyangeni letilishumi natimbili letendlulile ngusiphi sifundza losebentele kakhulu kuso kutfola inzuzo ngekulala?* | 01= Hhohho  02= Manzini  03= Shiselweni  04= Lubombo  05= Outside Swaziland  88= No Response  99= Don’t Know | \|__\|__\| |
|  | **3.08b**In the past 12 months, how often did you sell sex in a different region?  *Etinyangeni letilishumi natimbili letendlulile, utfole kangaphi inzuzo esifundzeni lesehlukile kuleso lohlale usebentela kuso?* | 00= Never  02= Sometimes  03= Frequently/Many times  88= No Response  99= Don’t know | \|__\|__\| |
|  | **3.08c**In the past 12 months, how often did you sell sex outside of the country of Swaziland?  *Etinyangeni letilishumi natimbili letendlulile, uyitfole kangakhi inzuzo ngekulala ngaphandle kwalelive?* | 00= Never  02= Sometimes  03= Frequently/Many times  88= No Response  99= Don’t know | \|__\|__\| |

| **3.09** | In the last 6 months, have you ever had vaginal or anal sex **without** a condom?  *Etinyangeni letisitfupha letendlulile uke walala yini ngembili noma ngemuva* ***ngaphandle*** *kwekusebentisa lijazi lemkhwenyana?* | 00= No  01= Yes  88= No Response  99= Don’t know | \|__\|__\| |
| --- | --- | --- | --- |
| **3.10** | How often have you used a condom when having vaginal or anal sex in the last 6 months?  *Ulisebentise kangakhi lijazi lemkhwenyana etinyangeni letisitfupha letendlulile nawulala ngembili noma ngemuva?* | 00= Never  01= Almost never  02= Sometimes  03= Almost always  04= Always  88= No Response  99= Don’t know | \|__\|__\| |
| **3.11a** | Do you share your earnings with a person who arranges clients for you or provides protection for you?  *Ingabe lenzuzo loyitfola kulomsebenti kukhona yini lomunye lomhlephulelako, lokungaba ngulokufunela emakhasimende noma lokuvikelako?* | 00= No**(Skip to Module 4)**  01= Yes  88= No Response**(Skip to Module 4)**  99= Don’t Know**(Skip to Module 4)** | \|__\|__\| |
|  | **3.11b** What benefits do you get from this relationship? (Mark all that apply)  *Utfolani wena kulobudlelwane bakho naye?*  *(Maka konkhe lokufanele)* | 00= No Benefit  01= Physical protection  02= Living arrangements  03= Food  04= Medical care  05=Drugs or alcohol  06=Emotional support  07= Other 🡪  _________________  88= No Response  99= Don’t know | \|__\|__\| |

|  | **3.11c** Are you free to end this relationship or stop paying this person?  *Ukhululekile yini kutsi ungabuyekela lobudlelwane noma ungabe usachubeka nekumbhadala lomuntfu?* | 00= No  01= Yes  02= Refuses to answer  88= No Response  99= Don’t know | \|__\|__\| |
| --- | --- | --- | --- |
|  |  |  |  |
|  |  |  |  |
| **Module 4 – CLIENTS**  “For the next questions, please think about men who give you money, favours or goods in exchange for sex. We will refer to these men as clients.”  *“Kulemibuto lelandzelako, ngiyacela ucabange ngebesilisa labakunika imali noma lokunye lokuyinzuzo ngoba ulale nabo. Utababita labesilisa sitsi ngemakhasimende.”* | | | |

| **4.00** | Counting **all** of your partners and clients, how many times in total did you have vaginal or anal sex in the last week?  *Nawubala bonkhe lotsandzana nabo nemakhasimende akho, ulele kangakhi ngembili nangemuva kuleliviki leliphelile (emalangeni lasikhombisa lendlulile).* | **Record number in box**  888= No Response **(Skip to 4.01)**  999= Don’t know | \|__\|__\|__\| |
| --- | --- | --- | --- |
| **4.01** | On how many of these times did you use a condom? I am talking about using condoms during the **entire**sex act, from start to finish.  *Kulokulala kwaleliviki leliphelile (emalangeni lasikhombisa lendlulile), ulisebentise kangaphi lijazi lemkhwenyana? Lapha ngikhuluma ngekulisebentisa nawucala kulala uze uyocedza.* | **Record number in box**  888= No Response  999= Don’t know | \|__\|__\|__\| |
| **4.02** | Where do you have sex with male clients the most?  **(Multiple answers acceptable)**  *Uvame kulala kuphi nemakhasimende akho esilisa sikhatsini lesinyenti?*  ***(Timphendvulo letinyenti tivumelekile)*** | 01= Private home  02= Bars/Clubs  03= Private Parties  04=Hotel  05= Street/Park  06= Private vehicle  07= Other:  _______________  88= No Response  99= Don’t know | \|__\|__\|  \|__\|__\|  \|__\|__\| |
| **4.03** | In the past 12 months have you used a mobile phone to meet up or arrange meetings with clients?  *Etinyangeni letilishumi natimbili letendlulile, ulusebentisile yini lucingo lwamahlalekhikhini kuhlela kuhlangana nemakhasimende akho?* | 00= No **(Skip to 4.03]**  01= Yes  88= No Response  99= Don’t Know | \|__\|__\| |
|  | **4.03a**How often do you use a mobile phone to meet up or arrange meetings with clients?  *Ulusebentisa kanganani lucingo lwamahlalekhukhwini kuhlelakuhlangana nemakhasimende akho?* | 01= Almost never  02= Sometimes  03= Almost always  04= Always  88= No Response  99= Don’t know | \|__\|__\| |
| **4.04** | In the past 12 months, have you used the Internet to look for or meet up with male clients?  *Etinyangeni letilishumi natimbili lenengcile, uke wasebentisa yini tekuchumana ngabongcondvomshini kufuna emakhasimende noma kuhlela kuhlangana nawo?* | 00= No **(Skip to 4.05)**  01= Yes  88= No Response  99= Don’t Know | \|__\|__\| |
|  | **4.04a** Which Internet site do you use the most?  *Bewusebentisa muphi umtfombo wekufuna?* | 01= Facebook  02=Other:________ | \|__\|__\| |
| **4.05a** | Do you receive cash money from clients?  *Uyayitfola yini inzuzo leyimali kumakhasimende akho?* | 1=Always  2=Very often  3=Sometimes  4=Almost never  5=Never | \|__\|__\| |
| **4.05b** | How much do you typically charge per client for vaginal sex with a condom?  *Uvame kulibita malini likhasimende nangabe ulele nalo ngembili nisebentise ikhondomu?* | **Record number in box**  888= No Response  999= Don’t know | \|__\|__\|__\| |
| **4.05c** | How much do you typically charge per client for vaginal sex without a condom?  *Uvame kulibita malini likhasimende nangabe ulele nalo ngembili ningakasebantisi ikhondomu?* | **Record number in box**  888= No Response  999= Don’t know | \|__\|__\| |
| **4.05d** | Do you receive goods or favours from clients?  *Uyayitfola yini inzuzo leyimphahla noma wentelwe lutfo tsite nge-makhasimende akho?* | 1=Always  2=Very often  3-Sometimes  4=Almost never  5=Never **(Skip to Module 4A)** | \|__\|__\| |
| **4.05e** | What types of goods or favours do you typically receive from clients?  *Luhlobo luni lwemphahla lephatsekako noma tintfo letinhle lowentelwa tona ngemakhasimende akho?* | 1=Food  2=Shelter  3=Transportation  4=Other:_______ | \|__\|__\| |

| **Module 4A: NEW CLIENTS**  “I’d like to start by asking you about your new clients. By new clients I mean clients that you had sex with only once or twice in your life.”  *“Ngitawutsandza kucala ngekukubuta ngemakhasimende akho lamasha. Emakhasimende lamasha ngulawo lolele nawo kanye nome kabili kuphela emphilweni yakho.”* |
| --- |

| **4.06a** | In the past 30 days, how many **new** clients have you had?  *Emalangeni langemashumi lamatsatfu lengcile, mangakhi emakhasimende* ***lamasha*** *lobe nawo?* | **Record number in the box**  888= No Response  999= Don’t know (**Skip to 4.11a)** | \|__\|__\|__\| |
| --- | --- | --- | --- |
|  | **4.06b** In the last 30 days how often did you use condoms (male or female) when having **vaginal** sex with **new** clients?Again, I am talking about how often you used condoms during the **entire** sex act, from start to finish.  *Emalangeni langemashumi lamatsatfu lendlulile, ulisebentise kangakhi lijazi lemkhwenyana (lebesilisa noma besifazane) nawulala nemakhasimende lamasha ngembili entfombini? (Lapha sikhuluma ngekutsi lelijazi lisentjetiswe kusukela nicala kulala kwaze kwaba sekugcineni)* | 00= Never**(Skip to 4.07a)**  01= Rarely  02= Sometimes  03= Most of the time  04= Always  88= No Response  99= Don’t know | \|__\|__\| |
|  | **4.06c**In the last 30 days, have you had **vaginal** sex with a new client when the condom has slipped off or broken?  *Emalangeni langemashumi lamatsatfu lengcile, uke wachubeka yini nekulala nelikhasimende lelisha ngembili lapho khona ikhondomu beseyidzabukile noma seyiphumile?* | 00= No  01= Yes  88= No Response  99= Don’t know | \|__\|__\| |

| **4.07a** | In the last 30 days, how often did you use condoms when having **anal** sex with new clients?  *Etinyangeni letilishumi natimbili letendlulile, uyisebentise kanganani ikhondomu nawulala nemakhasimende lamasha ngemuva?* | 00= Never**(Skip to 4.08a)**  01= Rarely  02= Sometimes  03= Most of the time  04= Always  05= NA – doesn’t practice anal sex  88= No Response  99= Don’t know | \|__\|__\| |
| --- | --- | --- | --- |
|  | **4.07b** In the last 30 days, have you had **anal** sex with a new client when the condom has slipped off or broken?  *Etinyangeni letilishumi natimbili letendlulile, uke walala yini nelikhasimende lelisha ngemuva ngesikhatsi ikhondomu seyidzabukile noma seyiphumile?* | 00= No  01= Yes  88= No Response  99= Don’t Know | \|__\|__\| |
| **4.08a** | In the last 30 days, how often did you use condoms when giving **oral** sex to new clients? **Read all options and provide single response.**  *Etinyangeni letilishumi natimbili letendlulile, ulisebentise kanganani lijazi lemkhwenyana nawulala ngemlomo nemakhasimende lamasha?*  ***(Mfundzele tonkhe timphendvulo bese ukunika yinye)*** | 00= Never  01= Rarely  02= Sometimes  03= Most of the time  04= Always  05= Doesn’t practice oral sex  88= No Response  99= Don’t know | \|__\|__\| |

| **4.09a** | In the last 30 days, how often did you talk to new clients about sexually transmitted infections and HIV?  *Emalangeni langemashumi lamatsatfu lengcile, ukhulume kangakhi nemakhasimende lamasha ngetifo letitsatselwana ngelicasi neHIV?* | 00= Never  01= Rarely  02= Sometimes  03= Most of the time  04= Always  88= No Response  99= Don’t know | \|__\|__\| |
| --- | --- | --- | --- |
| **4.10a** | Now think about the last time you hadvaginal or anal sex with a **new** client. Did you use a condom at that time?  *Ngicela ukhumbule ngesikhatsi ugcina kulala ngembili noma ngemuva nekhasimende lelisha. Walisentisa yini lijazi lemkhwenyana kuleso sikhatsi?* | 00= No  01= Yes**(Skip to 4.11)**  88= No Response  99= Don’t Know | \|__\|__\| |
|  | **4.10b** Why didn’t you use a condom at that time?  **[Do not read answers; permit multiple answers]**  *Awulisebentisanga ngani lijazi lemkhwenyana?* | 00= Not available  01= Client objected  02= I don’t like them  03= Used other method  04= Unnecessary/ safe partner  05= No time to find one  06= Forgot/didn’t think about it  07= Was offered more money not to use  08= Sex was forced  10= Other_______  88= No Response  99= Don’t know | \|__\|__\| |

| **Module 4B: REGUALR CLIENTS / REGULAR PAYING PARTNERS**  “Next I am going to ask you about your regular clients or regular paying partners. Regular clients or regular paying partners are those whom you have had sex with at least three times in your life and who have paid you for sex. This may also include long-time clients that help you pay for your living expenses or provide you with other financial support.”  *“Kulolokulandzelako ngitakubuta ngemakhasimende lejwayelekile akho noma labo labakubhadala njalonjalo. Emakhasimende lejwayelekile noma labo labakubhadala njalonjalo ngulabo lolele nabo lokungenani katsatfu futsi bakubhadala. Loku kufaka ekhatsi emakhasimende losobe nawo sikhatsi lesidze lakusita kutsi uhlangabete tidzingo takho tekuphila noma ngelusito lwemali.”* | | | |
| --- | --- | --- | --- |
|  |  |  |  |
| **4.11a** | In the last 30 days, how many **regular** clients have you had sex with?  *Emalangeni langemashumi lamatsatfu lendlulile, mangakhi emakhasimende akho lejwayelekile loke walala nawo?* | **Record number in the box**  888= No Response  999= Don’t know **(Skip to 4.17a)** | \|__\|__\|__\| |
| **4.12a** | In the last 30 days, how often did you use condoms (male or female) when having **vaginal** sex with these clients?  *Emalangeni langemashumi lamatsatfu lendlulile, ulisebentise kangakhi lijazi lemkhwenyana (lalabadvuna noma labasikati) nawulala ngembili nalamakhasimende akho lejwayelekile?* | 00= Never**(Skip to 4.13a)**  01= Rarely  02= Most of the time  03= Sometimes  04= Most of the time  05= Always  88= No Response  99= Don’t know | \|__\|__\| |
|  | **4.12b** In the last 30 days, have you had **vaginal** sex with a regular client when the condom has slipped off or broken?  *Kulamalanga langemashumi lamatsatfu lendlulile, uke wachubeka yini walala nelikhasimende lelejwayelekile ngembili noma lijazi lemkhwenyana seliphumile noma-ke selidzabukile?* | 00= No  01= Yes  88= No Response  99= Don’t know | \|__\|__\| |
| **4.13a** | In the last 30 days, how often did you use condoms when having **anal** sex with regular clients?  *Emalangeni langemashumi lamatsatfu lendlulile, ulisebentise kangakhi lijazi lemkhwenyana nawulala nelikhasimende lelejwayekile ngemuva?* | 00= Never**(Skip to 4.14a)**  01= Rarely  02= Sometimes  03= Most of the time  04= Always  05= Doesn’t practice anal sex  88= No Response  99= Don’t know | \|__\|__\| |
|  | **4.13b** In the last 30 days, have you had **anal** sex with a regular client when the condom has slipped off or broken?  *Emalangeni langemashumi lamatsatfu lendlulile, uke wachubeka yini nekulala ngemuva nelikhasimende lelejwayelekile noma lijazi lemkhwenyane selidzabukile noma-ke seliphumile?* | 00= No  01= Yes  88= No Response  99= Don’t know | \|__\|__\| |
| **4.14a** | *In the last 30 days, how often did you use condoms when giving* ***oral*** *sex to regular clients?****Read all options and provide single response.***  *Emalangeni langemashumi lamatsatfu lendlulile, ulisebentise kangakhi lijazi lemkhwenyana nawulala nelikhasimende lelejwayekile ngemlomo?* | 00= Never  01= Rarely  02= Sometimes  03= Most of the time  04= Always  05= Doesn’t practice anal sex  99= Don’t know | \|__\|__\| |
| **4.15a** | In the last 30 days, how often did you talk to regular clients about sexually transmitted infections and HIV?  *Emalangeni langemashumi lamatsatfu lendlulile, kukangakhi ukhuluma nemakhasimende lejwayelekile ngetifo tabogcunsula neligciwane lembulalave?* | 00= Never  01= Rarely  02= Sometimes  03= Most of the time  04= Always  88= No Response  99= Don’t know | \|__\|__\| |

| **4.16a** | Now think about the last time you had vaginal or anal sex with a **regular**client. Did you use a condom at that time?  *Ngicela ucabangisise ngesikhatsi ugcina kulala ngembili noma ngemuva nelikhasimende lelejwayelekile. Walisebentisa yini lijazi lemkhwenyana ngaleso sikhatsi?* | 00= No  01= Yes**(Skip to 4.17a)**  88= No Response  99= Don’t Know | \|__\|__\| |
| --- | --- | --- | --- |
|  | **4.16b** Why didn’t you use a condom at that time?  **[Do not read answers; permit multiple answers]**  *Awulisebentisanga ngani lijazi lemkhwenyana ngaleso sikhatsi?* | 00= Not available  01= Client objected  02= I don’t like them  03= Used other method  04= Unnecessary/ safe partner  05= No time to find one  06= Forgot/didn’t think about it  07= Was offered more money not to use  08= Sex was forced  10= Other_______  88= No Response  99= Don’t know | \|__\|__\| |

| ***Module 4C: NON-PAYING PARTNERS***  *“Next I am going to ask you about your non-paying partners. Non-paying partners are those who you have had sex with, but are not your clients and therefore do not pay you for sex. This may include partners you live with, boyfriends or girlfriends (who do not pay you) and husbands. This would also include partners you had sex with for pleasure, without any sort of payment.”*  *“Sengitakubuta imibuto macondzana nalovana nabo labangakubhadali. Laba ngulabo lolala nabo labangasiwo emakhasimende akho futsi labangakhokheli. Laba kungafaka ekhatsi lovana nabo futsi lohlala nabo; kungaba singani lesidvuna noma lesisikati noma-ke indvodza yakho. Kungabuye kube ngulabo lolele nabo ngoba ufuna kujabula, hhayi ngoba ufuna imali.”* | | | |
| --- | --- | --- | --- |
| **4.17a** | In the last 30 days, how many non-paying partners have you had sex with?  *Emalangeni langemashumi lamatsatfu lendlulile, bangakhi lovana nabo lolele nabo labangesiwo emakhasimende akho?* | **Record number in the box**  88= No Response  99= Don’t Know  **(If 00, skip to Module 5)** | \|__\|__\| |
|  | **4.17b** Are these non-paying partners men, women, or both?  *Laba lovana nabo ingabe bekungemadvodza, nome bafati, noma-ke emadvodza nebafati kanyekanye?* | 00= All men  01= All women  02= Both men and women  88= No Response  99= Don’t know | \|__\|__\| |
| **4.18a** | In the last 30 days, how often did you use condoms (male or female) when having **vaginal** sex with these partners?  *Emalangeni langemashumi lamatsatfu lendlulile, uyisebentise kangakhi ikhondomu (yabesilisa nome besifazane) nawulala ngembili nalaba lovana nabo?* | 00= Never (**Skip to 4.19a)**  01= Rarely  02= Sometimes  03= Most of the time  04= Always  77=Not applicable  88= No Response  99= Don’t know | \|__\|__\| |
|  | **4.18b** In the past 30 days, have you had **vaginal** sex with a non-paying partner when the condom has slipped off or broken?  *Emalangeni langemashumi lamatsatfu lendlulile, uke wachubeka yini walala ngembili nemuntfu lovana naye longakubhadali nome lijazi lemkhwenyane seliphumile nome-ke selidzabukile?* | 00= No  01= Yes  77=Not applicable  88= No Response  99= Don’t know | \|__\|__\| |
| **4.19a** | In the last 30 days, how often did you use condoms when having **anal** sex with non-paying partners?  *Emalangeni langemashumi lamatsatfu lendlulile, ulisebentise kangakhi lijazi lemkhwenyane nawulala ngemuva nalabo lovana nabo labangakubhadali?* | 00= Never **(Skip to 4.20)**  01= Rarely  02= Sometimes  03= Most of the time  04= Always  77=Not applicable  88= No Response  99= Don’t know | \|__\|__\| |
|  | **4.19b** In the last 30 days, have you had **anal** sex with a non-paying partner when the condom has slipped off or broken?  *Emalangeni langemashumi lamatsatfu lendlulile, uke wachubeka yini nekulala ngemuva ngesikhatsi lijazi lemkhwenyana lebenilisebentisa seliphumile noma selidzabukile naloyo lovana naye longabhadali?* | 00= No  01= Yes  77=Not applicable  88= No Response  99= Don’t know | \|__\|__\| |
| **4.20** | In the last 30 days, how often did you use condoms when practicing **oral** sex with non-paying partners? **Read all options and provide single response.**  *Emalangeni langemashumi lamatsatfu lendlulile, ulisebentise kangakhi lijazi lemkhwenyana ngesikhatsi ulala ngemlomo nalabo lovana nabo labangakubhadali?* | 00= Never  01= Rarely  02= Sometimes  03= Most of the time  04= Always  05= Doesn’t practice oral sex  88= No Response  99= Don’t know | \|__\|__\| |
| **4.21a** | Now think about the last time you had vaginal or anal sex with a **non-paying partner**. Did you use a condom that last time?  *Ngicela ucabangisise ngesikhatsi ugcina kulala ngembili noma ngemuva nemuntfu lovana naye longabhadali. Walisebentisa yini lijazi lemkhwenyana ngaleso sikhatsi?* | 00= No  01= Yes **(Skip to 4.22 )**  77=Not applicable  88= No Response  99= Don’t know | \|__\|__\| |
|  | **4.21b** Why didn’t you use a condom that time?  **[Do not read answers; permit multiple answers]**  *Wentiwa yini kutsi ungalisebentisi lijazi lemkhwenyane ngaleso sikhatsi?*  ***(Ungamfundzeli timphendvulo; timphendvulo letiningi tevumelekile)*** | 00= Not available  01= Too expensive  02= Partner objected  03= I don’t like them  04= Sex was forced/was raped  05= Unnecessary/ safe partner  06= No time to find one  07= Forgot/didn’t think about it  08= Other🡪________________  99= Don’t know | \|__\|__\| |
| **4.22** | In the last 30 days, how often did you talk to non-paying partners about sexually transmitted infections and HIV?  *Emalangeni langemashumi lamatsatfu lendlulile, kukangakhi lapho ukhulume khona ngetifo letitsatselwana ngelicansi noma iHIV nalovana nabo labangabhadali?* | 00= Never  01= Rarely  02= Sometimes  03= Most of the time  04= Always  99= Don’t know | \|__\|__\| |

**Module 5 – Anxiety and Depression**

“Now we are going to ask questions about symptoms of anxiety and depression. Please do not feel bad about answering as it will remain confidential.”

*“Nyalo-ke sesitawubuta imibuto lebuta ngetinkhomba tekukhatsateka. Siyacela kutsi ungesabi ngekuphendvula ngoba loko lokutawukhulunywa kutawugcinwa kuyimfihlo.”*

| **No.** | **Question** | **Coding** | **Response** |
| --- | --- | --- | --- |
| **5.00** | In the last 3 years, have you felt sad or had depressed mood for more than 2 weeks at a time?  *Uke wativela yini usesimeni sekukhatsateka lesingaba ngetulu kwemaviki lamabili (esikhatsini sisinye) kuleminyaka lemitsatfu leyengcile?* | 00= No  01= Yes  88= No Response  99= Don’t know | \|__\|__\| |
| **5.01a** | Have you ever felt like you wanted to end your life?  *Kuke kwenteka yini weva shengatsi ngabe kuphila uyakushiya, uyatibulala?* | 00= No **(Skip to Module 6)**  01= Yes  88= No Response  99= Don’t know | \|__\|__\| |
|  | **5.01b** Have you ever talked to a counselor about these feelings?  *Wakhona yini kukhulumisana nemeluleki ngalokutsi uva shengatsi ngabe uyatibulala?* | 00= No **(Skip to Module 6)**  01= Yes  88= No Response  99= Don’t know | \|__\|__\| |
|  | **5.01c** Did you tell the counselor that you sell sex?  *Wamtjela yini lomeluleki kutsi utfola inzuzo ngekulala?* | 00= No  01= Yes  88= No Response  99= Don’t know | \|__\|__\| |

**Module 6 – Knowledge, Attitudes, Behavior**

“Now we are going to ask you private questions. Please do not feel bad about answering as it will remain confidential”

*“Nyalo-ke sitakubuta imibuto leyimfihlo. Siyacela kutsi utivele ukhululekile ngoba tonkhe timphendvulo takho titawugcinwa tiyimfihlo.”*

| **No.** | **Question** | **Coding** | **Response** |
| --- | --- | --- | --- |
| **6.00** | What type of sex puts you most at risk for HIV infection?  *Nguluphi luhlobo lwekulalana lolukubeka engotini lenkhulu yekutfola ligciwane lembulalave?* | 01= Vaginal  02= Anal  03= Oral  04= All carry equal risk  88= No Response  99=Don’t Know | \|__\|__\| |
| **6.01** | Which is the safest lubricant to use during **vaginal** sex?  *Ngukuphi kwekugcobisa nanilalana ngembili lokuphephile lokungasentjetiswa?* | 00=Petroleum jelly or Vaseline  01=Body creams/fatty creams  02=Water-based lubricant  03=Saliva  04=No lubricant use  88= No Response  99=Don’t know | \|__\|__\| |
| **6.02** | Which is the safest lubricant to use during **anal** sex?  *Ngukuphi kwekugcobisa nanilalana ngemuva lokuphephile lokungasentjetiswa?* | 00=Petroleum jelly or Vaseline  01=Body creams/fatty creams  02=Water-based lubricant  03=Saliva  04=No lubricant use  88= No Response  99=Don’t know | \|__\|__\| |
| **6.03** | Can you get HIV from using a needle to inject illegal drugs?  *Ngekwati kwakho ungalitfola yini ligciwane lembulalave ngekusenbentisa inyalitsi yinye uma bantfu batijova ngetidzakamiswa?* | 00= No  01= Yes  88= No Response  99= Don’t Know | \|__\|__\| |
| **6.04** | In the past 12 months have you been tested for a sexually transmitted disease **other than HIV**(like syphilis, gonorrhea, chlamydia, or herpes)?  *Etinyangeni letilishumi natimbili letendlulile, uke wapotjolelwa yini tifo tabogcunsula ngaphandle kweHIV?* | 00= No  01= Yes  88= No Response  99= Don’t know | \|__\|__\| |
| **6.05** | In the past 12 months have you been told by a health care provider that you have a sexually transmitted disease **other than HIV** (like syphilis, gonorrhea or chlamydia, or herpes)?  *Etinyangeni letilishumi natimbili letendlulile, uke watjelwa yini betemphilo kutsi unetifo tabogcunsula ngaphandle kweHIV?* | 00= No  01= Syphilis  02= Gonorrhea  03=Chlamydia  04= Herpes  05= Other🡪  _____________  88= No Response  99= Don’t know | \|__\|__\|  \|__\|__\|  \|__\|__\| |
| **6.06a** | In the past 12 months, have you felt symptoms of a sexually transmitted disease including discharge from your vagina or sores on or around your vagina or anus?  *Etinyangeni letilishumi natimbili letendlulile, kukhona yini timphawu tabogcunsula loke wativa emtimbeni wakho – loku kungafaka ekhatsi kuphuma kungcola tsite ngembili noma tilondza noma emachuchumba ngembili noma ngemuva?* | 00= No **(Skip to 6.07)**  01= Yes  88= No Response  99= Don’t Know | \|__\|__\| |
|  | **6.06b** Was this infection treated at a clinic?  *Ingabe leto tifo watelaphisa yini kubetemphilo?* | 00= Yes, treated by doctor or nurse  01= No, self-treated  02= No, no treatment  88= No Response  99=Don’t Know | \|__\|__\| |
| **6.07** | Have you been tested for HIV in the last 12 months?  *Uke wahlolwa yini ingati mayelana neligciwane leHIV etinyangeni letilishumi natimbili letendlulile?* | 00= No  01= Yes, once  02=Yes, more than once  88= No Response  99= Don’t Know | \|__\|__\| |
| **6.08a** | Have you ever been told by a health care provider that you have HIV?  *Wake watjelwalwa yini betemphilo kutsi uneligciwane lembulalave?* | 00= No **(Skip to 6.09)**  01= Yes  88= No Response  99= Don’t know | \|__\|__\| |
|  | **6.08b** Are you getting treated for HIV?  *Kukhona yini lusito lwekunakekelwa ngekwelashwa lolutfolako mayelana naleligciwane leHIV?* | 00= No **(Skip to 6.09)**  01= Yes  88= No Response  99= Don’t know | \|__\|__\| |
|  | **6.08c** What treatment are you currently receiving?  *Lusito luni lolutfolako manje, nangabe lukhona?* | 00=Medication from health facility  01=Traditional herbs  88= No Response  99= Don’t know | \|__\|__\| |
| **6.09a** | Do you have access to condoms when you need them?  *Kulula kanganani kutfolakala kwelijazi lemkhwenyane ngalesikhatsi lolidzinga ngaso?* | 00= No Access  01= Difficult or little access  02= Somewhat difficult access  03= Somewhat easy access  04= Very easy access  88= No Response  99= Don’t know | \|__\|__\| |
|  | **6.09b** In general, do you buy condoms or get them for free?**Read all options and provide single response.**  *Empeleni, ingabe uvame kuwatsenga yini emakhondomu noma uwatfola mahhala?* | 01= Buy all of them **(Skip to 6.10a)**  02= Get all of them for free  03= Both buy and get them for free  88= No Response  99= Don’t know | \|__\|__\| |
|  | **6.09c** If you get them for free, where do you get them?  *Nangabe uwatfola mahhala, uwatfolaphi?* | 01= PSI  02= TASC  03= FLAS  04= Local clinic  05=Other🡪______  88= No Response  99= Don’t know | \|__\|__\| |
|  | **6.09d** In the past 30 days, how many condoms did you receive?It is okay to guess or estimate.  *Emalangeni langemashumi lamatsatfu lendlulile, ufinyelele kutfola mangakhi emakhondomu?*  *(Akunendzaba uma abekisa noma acagela)* | **Record number in the box**  88= No Response  99= Don’t know | \|__\|__\|\|__\|__\| |
|  | **6.09e**Was that quantity sufficient?  *Kulolowawatfola, ingabe enela yini?* | 00= No  01= Yes  88= No Response  99=Don’t Know | \|__\|__\| |
| **6.10a** | Do you use lubricants?  *Uyawasebentisa yini vele emafutsa ekugcobisa etindzaweni tekulala nangabe ulala nalomunye umuntfu?* | 00= No **(Skip to 6.11)**  01= Yes  88= No Response  99= Don’t know | \|__\|__\| |
|  | **6.10b** Do you have access to lubricants when you need them?  *Kulula kanganani kutfola kwekugcobisa nawulala ngesikhatsi ukudzinga?* | 00= No Access  01= Difficult or little access  02= Somewhat difficult access  03= Somewhat easy access  04= Very easy access  88= No Response  99= Don’t know | \|__\|__\| |
|  | **6.10c** Which type of lubricant do you generally use for vaginal/anal sex with men?  *Nguluphi luhlobo lwekwekugcobisa lolusebentisako ngembili noma ngemuva nawulala nebesilisa?* | 00=Petroleum jelly or Vaseline  01=Body creams/fatty creams  02=Water-based lubricant  03=Saliva  04=No lubricant use  88= No Response  99=Don’t know | \|__\|__\| |
| **6.11** | In the last 12 months, how worried would you say you have been about HIV/AIDS?  *Kuletinyanga letilishumi natimbili letendlulile, bewukhatsateke kanganani ngeligciwane lembulalave?* | 00= Very worried  01= Somewhat worried  02= Little worried  03= Not at all worried  88= No Response  99= Don’t know | \|__\|__\| |
| **6.12a** | In the last 12 months have you received any information on HIV prevention?  *Kuletinyanga letilishumi natimbili letendlulile, uke walutfola yini lwati ngekuvikela ligciwane lembulalave?* | 00= No  **(Skip to 6.13a)**  01= Yes  88= No Response  99= Don’t know | \|__\|__\| |
|  | **6.12b** If you have received information on prevention of HIV where did you get it?  *Walutfolaphi lolwati?* | 00= No information  01= Health Facility  02= Peer Educator/workshop  04= Internet  05= Friends  06= Other sex workers  07= Media  10= Other_______  88= No Response  99= Don’t know | \|__\|__\| |
| **6.13a** | In the last 12 months, have you participated in any talks or meetings related to HIV/AIDS?  *Kuletinyanga letilishumi natimbili letendlulile, uke wahlanganyela yini etinkhulumeni noma-ke emihlanganweni lemayelana ne HIV/AIDS?* | 00= No **(Skip to 6.14)**  01= Yes  88= No Response  99= Don’t know | \|__\|__\| |
|  | **6.13b** Who hosted these talks or meetings?  *Ngubani lebekabite leto tinkhulumo noma lemihlangano?* | 01= FLAS  02= TASC  03= PSI  04= Local clinic  05= Other________  88= No Response  99= Don’t know | \|__\|__\| |
| **6.14a** | In the last 12 months, have you participated in any talks or meetings related to HIV/AIDS with other sex workers?  *Kuletinyanga letilishumi natimbili letendlulile, uke wahlanganyela yini etinkhulumeni noma-ke emihlanganweni lemayelana ne HIV/AIDS kanye nalabanye labatfola imali ngekulala?* | 00= No **(Skip to 6.15)**  01= Yes  88= No Response  99= Don’t know | \|__\|__\| |
|  | **6.14b** Who hosted these talks or meetings with other sex workers?  *Ngubani lebekabite leto tinkhulumo noma lemihlangano lehlanganisa labatfola imali ngekulala?* | 01= FLAS  02= TASC  03= PSI  04= Local clinic  05= Other _______  88= No Response  99= Don’t know | \|__\|__\| |
| **6.15** | Have you ever been a part of a formal research project on sex workers in Swaziland before?  *Wake wacelwa yini kutsi ube yincenye yelucwaningo phambilini lwalabatfola inzuzo ngekulala lapha kaNgwane?* | 00= No  01= Yes  88= No Response  99= Don’t know | \|__\|__\| |
| **6.16a** | In the past 12 months have you injected illegal drugs?  *Kuletinyanga letilishumi natimbili letendlulile, uke watijova yini ngetidzakamizwa letingekho emtsetfweni?* | 00= No **(Skip to 6.17)**  01= Yes  88= No Response  99= Don’t know (**Skip to 6.17**) | \|__\|__\| |
|  | **6.16b** Have you shared needles with others?  *Kuke kwenteka yini wasebentisa imijovo lesentjetiswe ngulabanye?* | 00= No  01= Yes  88= No Response  99= Don’t know | \|__\|__\| |
| **6.17** | In the past 12 months have you used any non-injectable drug that was not prescribed?  These include marijuana, powdered cocaine, and narcotics.)  *Kuletinyanga letilishumi natimbili letendlulile,uke watisebentisa yini tidzakamizwa letingajovwa lebekungakashiwo kutsi tisebentise?*  *(Loku kungafaka ekhatsi insangu, cocaine, ne-narcotic)* | 00= No  01= Yes  88= No Response  99= Don’t know | \|__\|__\| |
| **6.18a** | In the past 30 days how many days did you drink at least one drink of alcohol?  *Emalangeni langemashumi lamatsatfu lendlulile, mangakhi emalanga lapho unatse khona lokungenani ingilazi yinye yetjwala?* | **Record number in box(If no alcohol use, write00and skip to Module 7)**  88= No Response  99= Don’t know | \|__\|__\| |
|  | **6.18b** In the past 30 days, when you did drink alcohol, how many drinks did you generally have in one sitting?  *Emalangeni langemashumi lamatsatfu lendlulile, bewunatsa tjwala lobunganani sikhatsi sisinye?* | **Record number in box**  88= No Response  99= Don’t know | \|__\|__\| |
|  | **6.18c** In the past 30 days, when you did drink alcohol, how often would you say you drank 5 or more drinks in one sitting?  *Emalangeni langemashumi lamatsatfu lendlulile,ngesikhatsi unatsa, kukangakhi lapho ungasho khona kutsi wanatsa tingilazi letingu 5 kuya etulu tetjwala?* | 01=Every time  02=Most of the time  03=Some of the time  04=Almost never  05=Never  88= No Response  99= Don’t know | \|__\|__\| |

**Module 7 – Condom Negotiation**

“I’m going to ask you several questions about using condoms and how difficult you may find it to negotiate condom use in different circumstances. For each situation, you can tell me if you find it very difficult, somewhat difficult, not very difficult, or not at all difficult.”

*“Ngitakubuta imibuto lembalwa mayelana nekusebentisa lijazi lemkhwenyana nebulukhuni lonabo ngekukhulumisana nalabanye ubelesele kutsi ayisentjetiswe etimeni letehlukene. Kuko konkhe lengitakubuta, utangitjela kutsi kube lukhuni kakhulu yini nome kube lukhunyana nome akukabi lukhuni kakhulu noma lokukanye akukabi lukhuni nhlobo.”*

| **No.** | Question | Coding | Response |
| --- | --- | --- | --- |
| **7.00** | How difficult is it for you to suggest using condoms with a client, even if it might make him think that you have a sexually transmitted disease?  *Kulukhuni kanganani kutsi uncome kutsi nisebentise ikhondomu nelikhasimende, noma ngabe sekubita kutsi acabange kutsi kungenteka kube ushiso kutsi unagcunsula?* | 01=Very difficult  02=Somewhat difficult  03=Not very difficult  04=Not at all difficult  99=Don't know | \|__\|__\| |
| **7.01** | How difficult is it for you to insist on condom use if a client does not want to use one?  *Kulukhuni kanganani kuwe kutsi ubelesele ngekusebentisa ikhondomu nangabe likhasimende lingafuni kuyisebentisa?* | 01=Very difficult  02=Somewhat difficult  03=Not very difficult  04=Not at all difficult  99=Don't know | \|__\|__\| |
| **7.02** | How difficult is it for you to continue to insist on condom use with a client even if he gets angry when you suggest it?  *Kulukhuni kanganani kutsi ubelesele ngekusebentisa ikhondomu noma ngabe likhasimende liyatfukutsela ngaloko?* | 01=Very difficult  02=Somewhat difficult  03=Not very difficult  04=Not at all difficult  99=Don't know | \|__\|__\| |
| **7.03** | How difficult is it for you to insist on condom use with a client when he has been drinking or using drugs?  *Kulukhuni kanganani kutsi ubelesele ngekusebentisa ikhondomu nangabe likhasimende linatsile nome lisebentise tidzakamizwa?* | 01=Very difficult  02=Somewhat difficult  03=Not very difficult  04=Not at all difficult  99=Don't know | \|__\|__\| |
| **7.04** | How difficult is it for you to insist on condom use with a client when you have been drinking or using drugs?  *Kulukhuni kanganani kutsi ubelesele ngekusebentisa ikhondomu nelikhasimende nangabe wena unatsile nome usebentise tidzakamizwa?* | 01=Very difficult  02=Somewhat difficult  03=Not very difficult  04=Not at all difficult  99=Don't know | \|__\|__\| |
| **7.05** | How difficult is it for you to insist on condom use with a client if you are sexually attracted to him?  *Kulukhuni kanganani kutsi ubelesele ngekusebentisa ikhondomu nangabe lelikhasimende utivela ulitsandza noma liyakukhanga kutekulala?* | 01=Very difficult  02=Somewhat difficult  03=Not very difficult  04=Not at all difficult  99=Don't know | \|__\|__\| |
| **7.06** | How difficult is it for you to insist on condom use with a client he offers you more money not to use a condom?  *Kulukhuni kanganani kutsi ubelesele ngekusebentisa ikhondomu nangabe likhasimende likunika imali lenyenti lentela kutsi ningayisebentisi ikhondomu?* | 01=Very difficult  02=Somewhat difficult  03=Not very difficult  04=Not at all difficult  99=Don't know | \|__\|__\| |
| **7.07** | How difficult is it for you to insist on condom use with a client with whom you haven’t always used condoms in the past?  *Kulukhuni kanganani kutsi ubelesele ngekusebentisa ikhondomu nangabe beningavami kuyisebentisa nalelo khasimende phambilini?* | 01=Very difficult  02=Somewhat difficult  03=Not very difficult  04=Not at all difficult  99=Don't know | \|__\|__\| |
| **7.08** | How difficult is it for you to insist on condom use with a client that you have known for a long time?  *Kulukhuni kanganani kutsi ubelesele ngekusebentisa ikhondomu nangabe likhasimende sewulati sikhatsi lesidze?* | 01=Very difficult  02=Somewhat difficult  03=Not very difficult  04=Not at all difficult  99=Don't know | \|__\|__\| |
| **7.09** | How difficult is it for you to insist on condom use with a client that you trust?  *Kulukhuni kanganani kutsi ubelesele ngekusebentisa ikhondomu nangabe lelo khasimende uyaletsemba?* | 01=Very difficult  02=Somewhat difficult  03=Not very difficult  04=Not at all difficult  99=Don't know | \|__\|__\| |
| **7.10** | How difficult is it for you to insist on condom use with a client that provides you with regular, economic support?  *Kulukhuni kanganani kutsi ubelesele ngekusebentisa ikhondomu nelikhasimende lelikusitako njalonjalo kutemnotfo?* | 01=Very difficult  02=Somewhat difficult  03=Not very difficult  04=Not at all difficult  99=Don't know | \|__\|__\| |
| **7.11** | How difficult is it for you to insist on condom use with a client that you care about?  *Kulukhuni kanganani kutsi ubelesele ngekusebentisa ikhondomu nelikhasimende loneluvelo nalo noma lolitsandzako?* | 01=Very difficult  02=Somewhat difficult  03=Not very difficult  04=Not at all difficult  88= No Response  99=Don't know | \|__\|__\| |
| **7.12** | How difficult is it for you to negotiate using a condom during oral sex with a client?  *Kulukhuni kanganani kugcizelela kusebentisa ikhondomu nangabe nilala ngemlomo nelikhasimende?* | 01=Very difficult  02=Somewhat difficult  03=Not very difficult  04=Not at all difficult  88= No Response  99=Don't know | \|__\|__\| |

**Module 8 - Social Capital**

“Now, I would like to ask you some questions about your social life and your sex worker colleagues.

I’m going to read some phrases and you can tell me if you strongly agree, mostly agree, mostly disagree, or strongly disagree.”

*“Nyalo-ke ngitawutsandza kukubuta ngekuphilisana kwakho kanye nalabanye nabo labatfola inzuzo ngekulala leninabo kulebhizinisi. Ngitawufundza imisho lemifisha utase uyangitjela kutsi uvumelana kanganani nayo.”*

| **No.** | Question | Coding | Response |
| --- | --- | --- | --- |
| **8.00** | You can count on your sex worker colleagues if you need to borrow money.  *Ungetsembela kulabanye bangani bakho labatfola inzuzo ngekulala nawufuna kuboleka imali.* | 00= Strongly disagree  01= Disagree  02= Agree  03= Strongly agree  88= No Response  99=Don't know | \|__\|__\| |
| **8.01** | You can count on your sex worker colleagues to accompany you to the doctor or hospital.  *Ungetsembela kulabanye labatfola inzuzo ngekulala losebenta nabo kutsi bangakuphekeletela nawuya kudokotela noma esibhedlela.* | 00= Strongly disagree  01= Disagree  02= Agree  03= Strongly agree  88= No Response  99=Don't know | \|__\|__\| |
| **8.02** | You can count on your sex worker colleagues if you need to talk about your problems.  *Ungetsembela kulabanye labatfola inzuzo ngekulala losebenta nabo nawufuna kukhuluma ngetinkinga takho.* | 00= Strongly disagree  01= Disagree  02= Agree  03= Strongly agree  88= No Response  99=Don't know | \|__\|__\| |
| **8.03** | In general, sex workers in the area where you live only worry about themselves.  *Ungasho kutsi lolosebenta nabo kulomsebenti wekutfola inzuzo ngekulala batikhatsalela bona bodvwa hhayi lomunye umuntfu.* | 00= Strongly disagree  01= Disagree  02= Agree  03= Strongly agree  88= No Response  99=Don't know | \|__\|__\| |
| **8.04** | You can count on your sex worker colleagues if you need somewhere to stay.  *Ungetsembela kulabanye lonabo kulomsebenti wekutfola inzuzo ngekulala uma udzinga indzawo yekuhlala.* | 00= Strongly disagree  01= Disagree  02= Agree  03= Strongly agree  88= No Response  99=Don't know | \|__\|__\| |
| **8.05** | You can count on your sex worker colleagues to help deal with a violent or difficult client.  *Ungetsembela kulabanye lonabo kulomsebenti wekutfola inzuzo ngekulala kutsi bangakusita nangabe likhasimende likulwisa noma likunika bulukhuni.* | 00= Strongly disagree  01= Disagree  02= Agree  03= Strongly agree  88= No Response  99=Don't know | \|__\|__\| |
| **8.06** | You can count on your sex worker colleagues to help you find other clients.  *Ungetsembela kulabanye lonabo kulomsebenti wekutfola inzuzo ngekulala kutsi bangakusita utfole emakhasimendi lamanyenti.* | 00= Strongly disagree  01= Disagree  02= Agree  03= Strongly agree  88= No Response  99=Don't know | \|__\|__\| |
| **8.07** | You can count on your sex worker colleagues to support the use of condoms.  *Ungetsembela kulabanye lonabo kulomsebenti wekutfola inzuzo ngekulala kutsi bangakusita ngekukhutsata kusebentisa ikhondomu.* | 00= Strongly disagree  01= Disagree  02= Agree  03= Strongly agree  88= No Response  99=Don't know | \|__\|__\| |
| **8.08** | The group of sex workers with whom you work is an integrated group.  *Ungasho kutsi laba lonabo kulomsebenti wekutfola inzuzo ngekulala bahlangene futsi bayevana.* | 00= Strongly disagree  01= Disagree  02= Agree  03= Strongly agree  88= No Response  99=Don't know | \|__\|__\| |
| **8.09** | In general the sex workers you work with are always arguing amongst each other.  *Ungasho kutsi laba lonabo kulomsebenti wekutfola inzuzo ngekulala bahlala baphikisana njalo.* | 00= Strongly disagree  01= Disagree  02= Agree  03= Strongly agree  88= No Response  99=Don't know | \|__\|__\| |
| **8.10** | You can trust the majority of other sex workers working in your area.  *Ungaletsemba linyenti lalabo lonabo kulomsebenti wekutfola inzuzo ngekulala lapho usebentela khona.* | 00= Strongly disagree  01= Disagree  02= Agree  03= Strongly agree  88= No Response  99=Don't know | \|__\|__\| |
| I am going to read you a list of different groups that you may be a part of. Please tell me in which of the following group activities do you participate in your personal life? If you do participate, would you consider yourself a member, active member, or group leader?  *Ngitawufundza luhla lwetinhlangotsi letitsite lokungenteka kutsi uyincenye yaletinye tato. Ngicela ungitjele kutsi nguluphi luhlangotsi lohlanganyela kulo. Nangabe uhlanganyela, ungasho yini kutsi ulilunga, noma ulilunga lelisebenta ngalokugcwele, noma ungumholi walelo cembu?* | | | |
|  | **8.11a** Church or religious groups  *Lisontfo noma kuletinye tinhlelo tenkholo* | 00=Do not participate  01=Member  02=Active member  03=Group Leader  88=No Response  99=Don't know | \|__\|__\| |
|  | **8.11b** Clubs (sports, students)  *Emacenjini (etemidlalo, ebafundzi)* | 00=Do not participate  01=Member  02=Active member  03=Group Leader  88=No Response  99=Don't know | \|__\|__\| |
|  | **8.11c** Cultural activities (dance, music,etc )  *Kutemasiko (kugidza, umculo, nalokunye)* | 00=Do not participate  01=Member  02=Active member  03=Group Leader  88=No Response  99=Don't know | \|__\|__\| |
|  | **8.11d**Community Activities (type)  *Imisebenti leyentiwa emangweni.* | 00=Do not participate  01=Member  02=Active member  03=Group Leader  88=No Response  99=Don't know | \|__\|__\| |
| **8.11e** | Others____________________________________  _________________________________________  *Lokunye:_______________________________________* | Document other activities mentioned | \|__\|__\| |
| **8.12** | In the past 12 months, how often have you participated in a meeting, march, rally, or gathering to promote the rights of sex workers?  *Etinyangeni letilishumi natimbili letendlulile, uhlanganyele kangakhi emihlanganweni, emishucweni, nasemicimbini lekhutsata emalungelo ebantfu labatfola inzuzo ngekulala?* | 00=Never  01=Once  02=A couple of times  03=Frequently | \|__\|__\| |
| **8.13** | In the past 12 months, how often have you gotten together with other sex workers to speak with government officials or political leaders to address a problem or common issue facing sex workers?  *Etinyangeni letilishumi natimbili letendlulile, uhlangane kangakhi nalabanye lonabo kulebhizinisi kutsi nikhulumisane netisebenti tahulumende nome baholi betembusave kutsi kukhulunyiswe indzaba tsite lebhekene nebantfu labatfola inzuzo ngekulala?* | 00=Never  01=Once  02=A couple of times  03=Frequently | \|__\|__\| |
| **8.14** | In the past 12 months, how often have you joined together with other sex workers to address a common problem facing sex workers?  *Etinyangeni letilishumi natimbili letendlulile, uhlanganyele kangakhi nalabanye labakulebhizinisi kutsi nicatulule inkinga tsite lebenibhekene nayo?* | 00=Never  01=Once  02=A couple of times  03=Frequently | \|__\|__\| |
| **8.15** | In the past 12 months, how often have you participated in an HIV prevention organization or sex worker rights group?  *Etinyangeni letilishumi natimbili letendlulile, uhlanganyele kangakhi enhlanganweni lefundzisa ngekuvikela iHIV noma-ke ecenjini lelilwela emalungelo ebantfu labatfola inzuzo ngekulala?* | 00=Never  01=Once  02=A couple of times  03=Frequently | \|__\|__\| |

**Module 9—Reproductive Health**

"Now I am going to ask you some questions about your reproductive health. Please do not feel bad about answering as it will remain confidential”

*“Lemibuto lelandzelako imayelana nekutfola bantfwana. Ngicela kutsi ungesabi nawuphendvula ngoba konkhe kutawugcinwa kuyimfihlo.”*

| **No.** | **Question** | **Coding** | **Response** |
| --- | --- | --- | --- |
| **9.00** | Have you ever been pregnant, including pregnancy that ended in miscarriage or abortion?  *Wake wakhulelwa yini phambilini? Loku kungaba sisu lesakonakalela noma lowagcina usikhiphile.* | 00= No **(Skip to 9.03)**  01= Yes  88= No Response  99= Don’t know | \|__\|__\| |
| **9.01** | Have you ever had a pregnancy that was unwanted? What I mean by that is, **at the time when you found out you were pregnant**,you did not want to be pregnant then.  *Wake wakhulelwa yini wativela ungafuni kuba naleso sisu?* | 00= No  01= Yes  88= No Response  99= Don’t know | \|__\|__\| |
| **9.02** | Have you ever had an abortion?  *Wake wasikhipha yini sisu?* | 00= No **(Skip to 9.04)**  01= Yes  88= No Response  99= Don’t know | \|__\|__\| |
| **9.03** | How many abortions have you ever had?  *Watikhipha kangaphi tisu?* | **Record number in box**  88= No Response  99= Don’t know | \|__\|__\| |
| **9.04** | Are you currently trying to get pregnant?  *Uyetama yini kutsi ukhulelwe nyalo?* | 00= No  01= Yes  02=Unsure  88= No Response  99= Don’t know | \|__\|__\| |
| **9.05** | How important is it for you to avoid getting pregnant now? **[Read each response option.]**  *Kumcoka kanganani kutsi ungagwema kukhulelwa nyalo?*  ***(Mfundzele timphendvulo)*** | 00=Not important at all  01=Not so important  02=Somewhat important  03=Very important | \|__\|__\| |
| **9.06** | I am going to read you a list of different methods that women may use to prevent pregnancy. For each method I mention, please tell me if you are currently using it. Some women use more than one method, so you can say more than one. **[Read each method aloud and check all that apply.]**  *Ngitawufundza luhla lwetindlela letehlukene tekuhlela kutsi make angakhulelwa. Kuyo yonkhe indlela lengiyibalako ngicela ungitjele kutsi uyayisebentisa yini. Kungenteka kutsi usebentisa lengetulu kwayinye, ngicela usho.* | 01=Female sterilization  02=Pill  03=IUD  04=Injectable  05=Implant  06=Male condom  07=Female condom  08= Diaphragm  09=Foam/jelly  10=Rhythm method  11=Withdrawal  12=Other_________ | \|__\|__\|  \|__\|__\|  \|__\|__\|  \|__\|__\|  \|__\|__\| |
| **9.07** | Have you ever used emergency contraception to prevent pregnancy after having unprotected sex? Emergency contraception is also known as the “morning after pill.”  *Uke walisebentisa yini liphilisi lelinatfwa ekuseni nangabe ulele ngaphandle kwekutivikela?* | 00= No  01= Yes  88= No Response  99= Don’t know | \|__\|__\| |

END

***NOTE: Affix label with identical unique ID to specimen collected***

0 = No

1 = Yes

**Sent to Expert Client?**

0 = No

1 = Yes

**Given condoms and lubrication?**

0 = No

1 = Yes

**Given HIV/AIDS material?**
