# Supplementary material for: Prevalence and correlates of anal intercourse among female sex workers in eSwatini
Source: PLoS One. 2020 Feb 11;15(2):e0228849. doi: 10.1371/journal.pone.0228849 (PMC7012411; doi:10.1371/journal.pone.0228849)
Supplement: S1 Table — (DOCX) [file pone.0228849.s002.docx]

**S1 Table:** Characteristics and behaviours included in the multivariable analysis, stratified by interviewer. The intraclass correlation coefficient (ICC) was used to measure potential interviewer effects between responses to individual interviewers

|  |  |  | **Interviewer 1**  **N=86** | | **Interviewer 2**  **N=57** | | **Interviewer 3**  **N= 89** | | **Interviewer 4**  **N= 88** | | **ICC**^†^ |
| --- | --- | --- | --- | --- | --- | --- | --- | --- | --- | --- | --- |
|  | |  | **Male, 30, HIV management** | | **Male, 31, HIV management** | | **Female, 25, psychology** | | **Female, 27, psychology** | |  |
| **Variable** | **Category** | **N** | **n** | **%** | **n** | **%** | **n** | **%** | **n** | **%** |  |
| **Outcomes** |  |  |  |  |  |  |  |  |  |  |  |
| Any AI in past month | No | 191 | 34 | 40% | 33 | 58% | 56 | 63% | 68 | 77% | 0.096 |
|  | Yes | 129 | 52 | 61% | 24 | 42% | 33 | 37% | 20 | 23% |  |
| Any AI with inconsistent condom use in past month^¶^ | No | 214 | 37 | 44% | 43 | 77% | 59 | 66% | 75 | 85% | 0.140 |
|  | Yes | 104 | 48 | 57% | 13 | 23% | 30 | 34% | 13 | 15% |  |
| **Personal characteristics** |  |  |  |  |  |  |  |  |  |  |  |
| Age | <26 years | 167 | 55 | 64% | 24 | 42% | 46 | 52% | 42 | 48% | 0.007 |
|  | 26+ | 153 | 31 | 36% | 33 | 58% | 43 | 48% | 46 | 52% |  |
| Highest level of education | Primary or lower | 104 | 28 | 33% | 20 | 35% | 23 | 26% | 33 | 38% | 0.000 |
|  | Some secondary or higher | 216 | 58 | 67% | 37 | 65% | 66 | 74% | 55 | 63% |  |
| Grew up | Urban | 157 | 45 | 54% | 33 | 59% | 47 | 53% | 32 | 36% | 0.000 |
|  | Rural | 148 | 39 | 46% | 22 | 39% | 39 | 44% | 48 | 55% |  |
|  | Foreign country | 12 | 0 | 0% | 1 | 2% | 3 | 3% | 8 | 9% |  |
| Number of dependents supported by sex work | 0-2 | 153 | 48 | 56% | 26 | 46% | 39 | 44% | 40 | 45% | 0.001 |
|  | 3+ | 167 | 38 | 44% | 31 | 54% | 50 | 56% | 48 | 55% |  |
| **Individual behaviour** |  |  |  |  |  |  |  |  |  |  |  |
| Number of sex acts/week | <5 | 162 | 50 | 60% | 33 | 61% | 44 | 50% | 35 | 40% | 0.026 |
|  | 5+ | 152 | 34 | 41% | 21 | 39% | 44 | 50% | 53 | 60% |  |
| Condom use at last sex with new or regular client^‡^ | Condom used | 242 | 71 | 84% | 39 | 71% | 68 | 76% | 64 | 73% | 0.000 |
|  | Condomless | 75 | 14 | 17% | 16 | 29% | 21 | 24% | 24 | 27% |  |
| Number of new clients/month | <5 | 183 | 45 | 58% | 40 | 74% | 51 | 59%­ | 47 | 53% | 0.015 |
|  | 5+ | 123 | 33 | 42% | 14 | 26% | 35 | 41% | 41 | 47% |  |
| Number of regular clients/month (5 NAs) | <7 | 184 | 40 | 48% | 37 | 65% | 59 | 68% | 48 | 55% | 0.026 |
|  | 7+ | 131 | 43 | 52% | 20 | 35% | 28 | 32% | 40 | 45% |  |
| Number of non-paying partners/month | 0 or 1 | 206 | 47 | 55% | 40 | 70% | 60 | 67% | 59 | 67% | 0.006 |
|  | 2+ | 113 | 38 | 45% | 17 | 30% | 29 | 33% | 29 | 33% |  |
| Any drug use/year | No | 207 | 55 | 64% | 40 | 70% | 54 | 64% | 58 | 66% | 0.028 |
|  | Yes | 108 | 31 | 36% | 17 | 30% | 30 | 36% | 30 | 34% |  |
| **Social discrimination/violence** | | | |  |  |  |  |  |  |  |  |
| Ever blackmailed | No | 210 | 53 | 62% | 43 | 75% | 63 | 71% | 51 | 58% | 0.014 |
|  | Yes | 110 | 33 | 38% | 14 | 25% | 26 | 29% | 37 | 42% |  |
| Ever physically or verbally harassed | No | 125 | 48 | 56% | 24 | 42% | 31 | 35% | 22 | 25% | 0.069 |
|  | Yes | 195 | 38 | 44% | 33 | 58% | 58 | 65% | 66 | 75% |  |
| Ever raped since age 18 | No | 180 | 51 | 63% | 31 | 54% | 48 | 58% | 50 | 61% | 0.009 |
|  | Yes | 123 | 30 | 37% | 26 | 46% | 35 | 42% | 32 | 39% |  |
| Ever afraid to access health services | No | 180 | 48 | 56% | 34 | 60% | 55 | 62% | 43 | 49% | 0.001 |
|  | Yes | 140 | 38 | 44% | 23 | 40% | 34 | 38% | 45 | 51% |  |
| Ever afraid to walk in public places | No | 167 | 51 | 59% | 30 | 53% | 50 | 56% | 36 | 41% | 0.016 |
|  | Yes | 153 | 35 | 41% | 27 | 47% | 39 | 44% | 52 | 59% |  |
| Social cohesion score^§^ | High | 157 | 35 | 47% | 21 | 39% | 51 | 61% | 34 | 39% | 0.034 |
|  | Low | 141 | 39 | 53% | 33 | 61% | 32 | 39% | 53 | 61% |  |
| **Knowledge and information access** | | | | |  |  |  |  |  |  |  |
| Knowledge of type of sex with highest transmission risk | Anal | 34 | 13 | 15% | 6 | 11% | 6 | 7% | 9 | 10% | 0.001 |
|  | Other | 286 | 73 | 85% | 51 | 90% | 83 | 93% | 79 | 90% |  |
| Tested for STIs/year | No | 232 | 54 | 63% | 50 | 88% | 67 | 75% | 61 | 69% | 0.035 |
|  | Yes | 88 | 32 | 37% | 7 | 12% | 22 | 25% | 27 | 31% |  |
| Received information on HIV prevention/year | No | 45 | 15 | 18% | 8 | 14% | 18 | 21% | 4 | 5% | 0.031 |
|  | Yes | 272 | 70 | 82% | 49 | 86% | 70 | 80% | 83 | 95% |  |

AI=anal intercourse, ICC=intraclass correlation coefficient, STI=sexually transmitted infection.

^†^The ICC measures the percentage of the total variance for a particular question that is attributable to the interviewer. A zero value represents no interviewer effect but as some variation across interviewers is to be expected, acceptable values are considered to be <0.07, with values above implying that respondents' answers were influenced by characteristics or behaviours of the interviewer when answering a survey question[30]. Two variables which were to be entered into the multivariate model were removed as the ICC indicated substantial interviewer effects (ICC>0.15) although they were not stigmatised topics (condom negotiation and social participation), which suggests that they were badly measured. The ICC measures differences between each individual interviewer.

^¶^AI practice with inconsistent condom use is defined as reporting having used condoms ‘most of the time’, ‘sometimes’, ‘rarely’ or ‘never’ during AI in the past month.

^‡^Condomless last sex with new or regular client was derived from two questions on condom use at last sex with new and regular clients separately, with condomless sex defined as reporting no condom use with either or both of these client types.

^§^The social cohesion score comprises of a series of questions on relationship with other FSW and was measured using a scale developed for use among FSW in Brazil[63]. Participants were asked to rate their agreement or disagreement with nine statements relating to mutual aid, support and trust among their peers, such as being able to count on colleagues to support the use of condoms and to help deal with violent or difficult clients. For analysis, the nine items were summed and the scores dichotomised at the median.
