# Supplementary material for: Prevalence and correlates of anal intercourse among female sex workers in eSwatini
Source: PLoS One. 2020 Feb 11;15(2):e0228849. doi: 10.1371/journal.pone.0228849 (PMC7012411; doi:10.1371/journal.pone.0228849)
Supplement: S2 Table — (DOCX) [file pone.0228849.s003.docx]

**S2 Table:** Demographic, behavioural and structural determinants of practicing anal intercourse with inconsistent condom use in the past month with any partner, among the whole sample of Swazi female sex workers (stratified by practice of AI with inconsistent condom use, and univariate and multivariable logistic regression with clustered standard errors). Stratified analysis shows crude data, logistic regression results are from models with imputed missing data.

|  |  |  | **AI with inconsistent condom use/past month^¶^** | | **No AI with inconsistent condom use/past month** | | **Univariate** | | **Multivariable^†^** | |
| --- | --- | --- | --- | --- | --- | --- | --- | --- | --- | --- |
| **Variable** | **Categories** | **N** | **n** | **%** | **n** | **%** | **OR** | **95% CI** | **aOR** | **95% CI** |
| **Personal characteristics** |  |  |  |  |  |  |  |  |  |  |
| Age | <26 years | 145 | 48 | 46 | 97 | 45 | Ref | - | Ref | - |
|  | 26+ | 173 | 56 | 54 | 117 | 55 | 0.91 | 0.56-1.5 | 1.06 | 0.57-1.98 |
| Highest level of education | Primary or lower | 104 | 35 | 28 | 69 | 36 | Ref | - | Ref | - |
|  | Some secondary or higher | 214 | 92 | 72 | 122 | 64 | 1.49 | 0.87-2.56 | 1.87* | 1.01-3.47 |
| Grew up | Urban | 155 | 47 | 45 | 108 | 51 | Ref | - | Ref | - |
|  | Rural | 148 | 52 | 50 | 96 | 46 | 1.31 | 0.83-2.09 | 1.94* | 1.12-3.38 |
|  | Foreign country | 12 | 5 | 5 | 7 | 3 | 1.66 | 0.54-5.07 | 7.11** | 1.71-29.49 |
| Number of dependents supported by sex work | 0-2 | 152 | 52 | 50 | 114 | 53 | Ref | - | Ref |  |
|  | 3+ | 166 | 52 | 50 | 100 | 47 | 0.88 | 0.55-1.40 | 0.75 | 0.43-1.32 |
| **Individual behaviour** |  |  |  |  |  |  |  |  |  |  |
| Number of sex acts/week  (5 NAs) | <5 | 199 | 77 | 76 | 122 | 58 | Ref | - | Ref | - |
|  | 5+ | 114 | 25 | 25 | 89 | 42 | 0.45** | 0.26-0.77 | 0.66 | 0.34-1.28 |
| Number of new clients/month | <5 | 182 | 73 | 75 | 109 | 52 | Ref | - | Ref | - |
|  | 5+ | 123 | 24 | 25 | 99 | 48 | 0.34*** | 0.20-0.58 | 0.31** | 0.15-0.67 |
| Number of regular clients/month | <7 | 183 | 63 | 62 | 120 | 57 | Ref | - | - |  |
|  | 7+ | 130 | 39 | 38 | 91 | 43 | 0.88 | 0.56-1.37 | 1.37 | 0.75-2.42 |
| Number of non-paying partners/month | 0 or 1 | 204 | 58 | 56 | 146 | 68 | Ref | - | Ref | - |
|  | 2+ | 113 | 45 | 44 | 68 | 32 | 1.66* | 1.04-2.64 | 1.45 | 0.80-2.63 |
| Any drug use/year | No | 205 | 61 | 60 | 144 | 68 | Ref | - | Ref | - |
|  | Yes | 108 | 41 | 40 | 67 | 32 | 1.39 | 0.85-2.27 | 1.43 | 0.79-2.59 |
| **Social discrimination/violence** | |  |  |  |  |  |  |  |  |  |
| Ever blackmailed | No | 208 | 73 | 51 | 135 | 63 | Ref | - | - |  |
|  | Yes | 110 | 31 | 49 | 79 | 37 | 0.74 | 0.45-1.23 | 0.70 | 0.34-1.46 |
| Ever physically or verbally harassed | No | 123 | 39 | 38 | 84 | 39 | Ref | - | Ref | - |
|  | Yes | 195 | 65 | 63 | 130 | 61 | 1.08 | 0.67-1.72 | 1.86* | 1.02-3.64 |
| Ever raped since age 18 | No | 179 | 49 | 51 | 130 | 63 | Ref | - | Ref | - |
|  | Yes | 122 | 47 | 49 | 75 | 37 | 1.70* | 1.01-2.88 | 1.94 | 0.93-2.06 |
| Ever afraid to access health services | No | 178 | 50 | 48 | 128 | 60 | Ref | - | Ref | - |
|  | Yes | 140 | 54 | 52 | 86 | 40 | 1.61 | 0.95-2.58 | 2.18** | 1.16-4.10 |
| Ever afraid to walk public places | No | 165 | 63 | 61 | 102 | 48 | Ref | - | Ref | - |
|  | Yes | 153 | 41 | 39 | 112 | 52 | 0.59* | 0.37-0.97 | 0.46* | 0.21-0.99 |
| Social cohesion score^§^­ | No | 140 | 46 | 50 | 94 | 46 | Ref | - | Ref | - |
|  | Yes | 156 | 47 | 50 | 109 | 54 | 0.85 | 0.52-1.40 | 0.80 | 0.43-1.49 |
| **Knowledge, information and services access** | |  |  |  |  |  |  |  |  |  |
| Knowledge of type of sex with highest transmission risk | Anal | 33 | 13 | 13 | 20 | 9 | Ref | - | Ref | - |
|  | Other | 285 | 91 | 88 | 194 | 91 | 0.72 | 0.34-1.52 | 0.53 | 0.20-1.38 |
| Tested for STI/past year | Yes | 232 | 76 | 73 | 58 | 27 | Ref | **-** | Ref | - |
|  | No | 86 | 28 | 27 | 156 | 73 | 0.96 | 0.58-1.60 | 1.68 | 0.90-3.13 |
| Received information on HIV prevention/past year | Yes | 270 | 87 | 84 | 183 | 86 | Ref | - | Ref | - |
|  | No | 45 | 16 | 16 | 29 | 14 | 1.16 | 0.59-2.28 | 1.20 | 0.55-2.15 |

AI=anal intercourse, aOR=adjusted odds ratio, OR=odds ratio, STI=sexually transmitted infection, 95%CI=95% confidence interval, Ref=reference level. *p<0.05, **p<0.01, ***p<0.001.

^†^ Multivariable results are mutually adjusted for all variables listed in this table. In addition to the variables listed, interviewer was entered into the model as a dummy variable in order to control for its potential confounding effect.

^¶^ Practice of AI with inconsistent condom use is defined as reporting anything other than ‘always’ having used condoms during AI in the past month with any partner type (i.e. using condoms most of the time, sometimes, rarely or never).

^‡^Condom use at most recent sex with new or regular clients was derived from two questions on condom use at last sex with new and regular clients separately, with condomless sex defined as reporting no condom use during AI or VI with either or both of these client types.

^§^ Social cohesion is an index comprised of a series of questions on relationship with other FSW. For more information, see S4 Table footnotes
