# Supplementary material for: Prevalence and correlates of anal intercourse among female sex workers in eSwatini
Source: PLoS One. 2020 Feb 11;15(2):e0228849. doi: 10.1371/journal.pone.0228849 (PMC7012411; doi:10.1371/journal.pone.0228849)
Supplement: S1 Fig — (DOCX) [file pone.0228849.s004.docx]

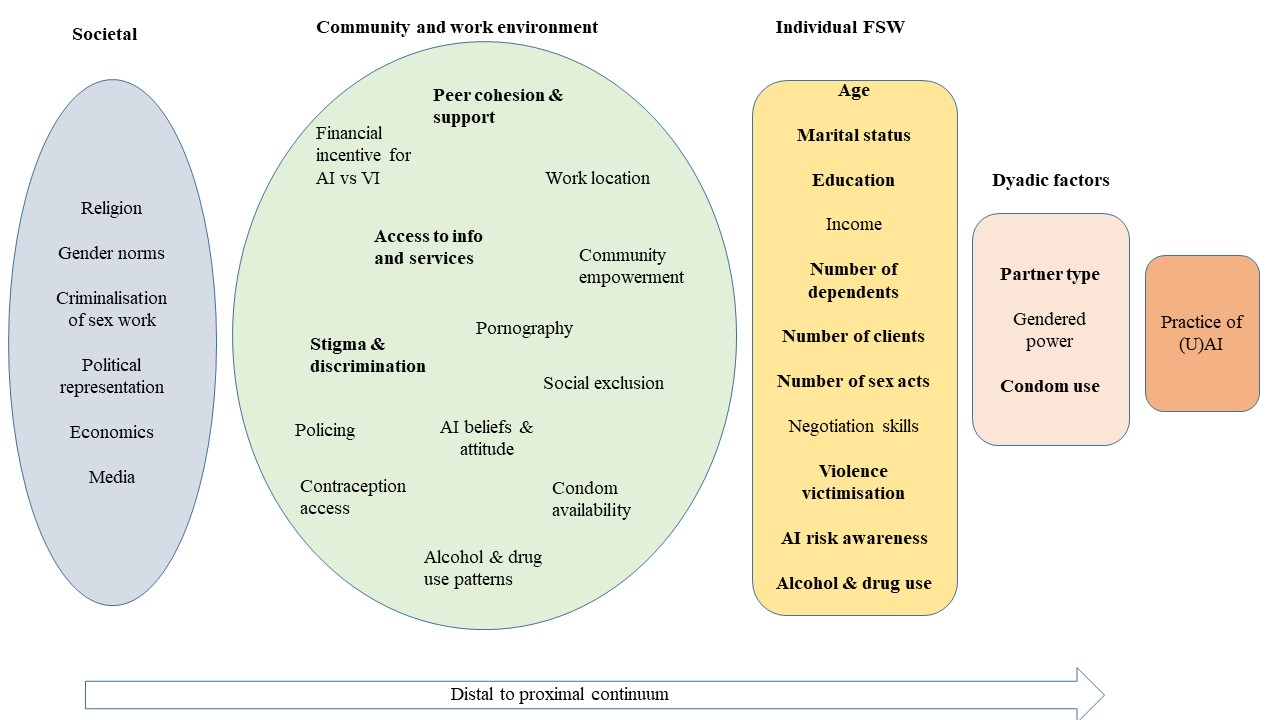


**S1 Figure**: A conceptual framework of anal intercourse practice among female sex workers. Bold font indicates covariates of interest for which data were available in this dataset.
