# Supplementary material for: Prevalence and correlates of anal intercourse among female sex workers in eSwatini
Source: PLoS One. 2020 Feb 11;15(2):e0228849. doi: 10.1371/journal.pone.0228849 (PMC7012411; doi:10.1371/journal.pone.0228849)
Supplement: S2 Fig — (DOCX) [file pone.0228849.s005.docx]

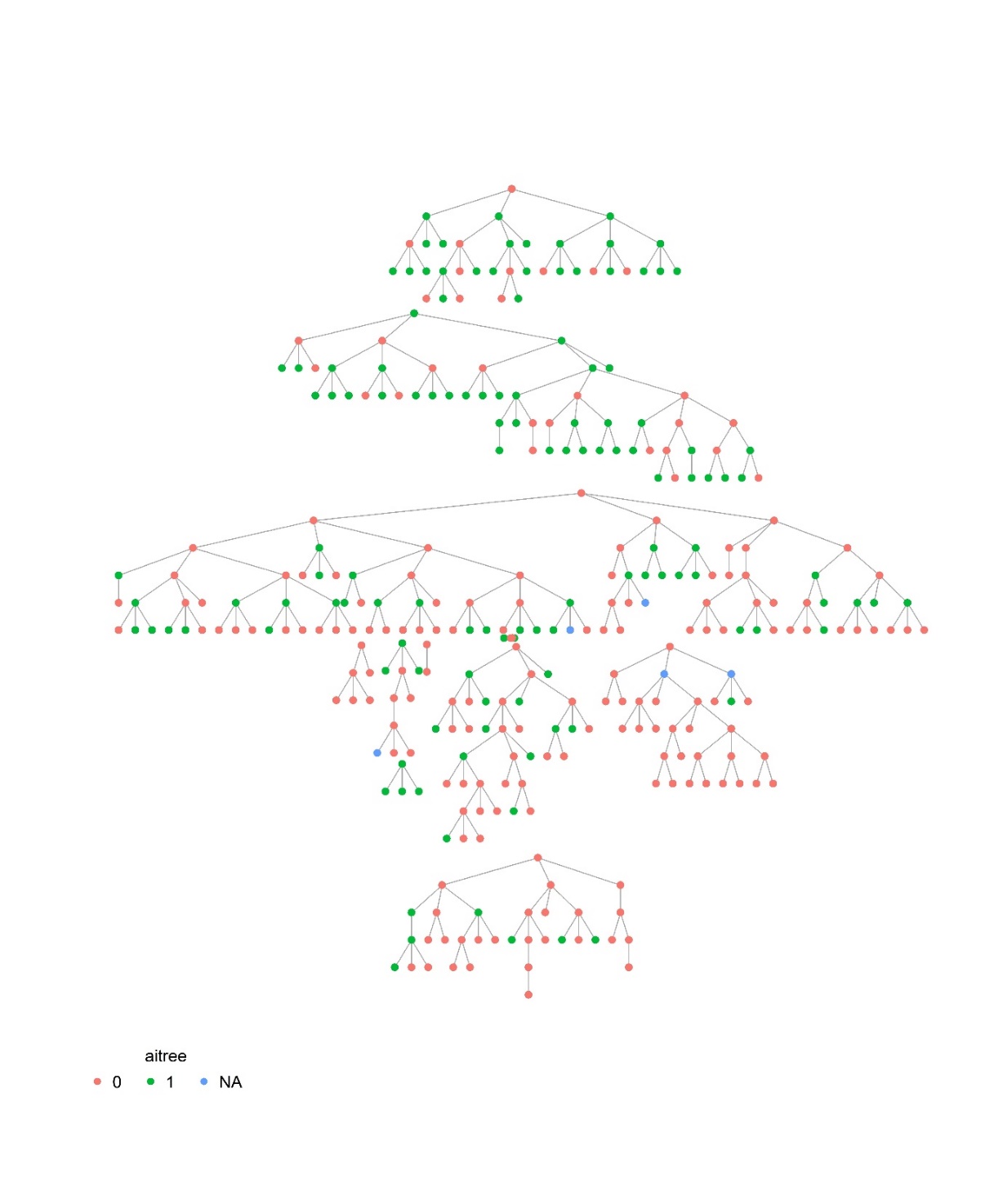


**S2 Figure:** RDS recruitment network tree.

Dots which are unconnected to dots above represent seeds. Nine of 14 total seeds recruited other FSW. FSW reporting AI practice in the past month are represented by green dots, FSW who report no AI practice by red dots. FSW with missing values for AI practice are represented by blue dots
